# Stochastic Generalized Active Space Self-Consistent Field: Theory and Application

**DOI:** 10.1021/acs.jctc.1c00936

**Published:** 2021-12-13

**Authors:** Oskar Weser, Kai Guther, Khaldoon Ghanem, Giovanni Li Manni

**Affiliations:** † Max-Planck-Institute for Solid State Research, Stuttgart, 70569, Germany; ‡ RIKEN Center for Computational Science, 7-1-26 minatojima-minami, Chuo Kobe 650-0047, Japan

## Abstract

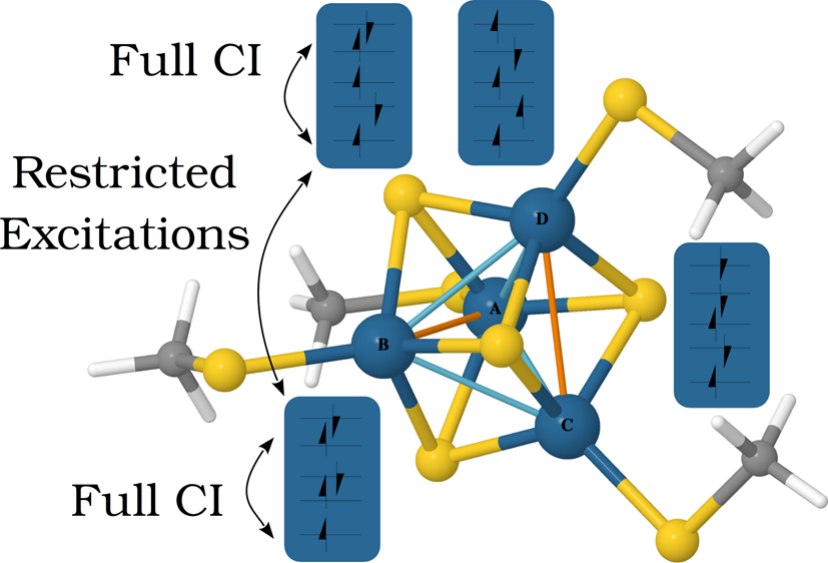

An algorithm to perform stochastic generalized active space calculations, Stochastic-GAS, is presented, that uses the Slater determinant based FCIQMC algorithm as configuration interaction eigensolver. Stochastic-GAS allows the construction and stochastic optimization of preselected truncated configuration interaction wave functions, either to reduce the computational costs of large active space wave function optimizations, or to probe the role of specific electron correlation pathways. As for the conventional GAS procedure, the preselection of the truncated wave function is based on the selection of multiple active subspaces while imposing restrictions on the interspace excitations. Both local and cumulative minimum and maximum occupation number constraints are supported by Stochastic-GAS. The occupation number constraints are efficiently encoded in precomputed probability distributions, using the precomputed heat bath algorithm, which removes nearly all runtime overhead of GAS. This strategy effectively allows the FCIQMC dynamics to *a priori* exclude electronic configurations that are not allowed by GAS restrictions. Stochastic-GAS reduced density matrices are stochastically sampled, allowing orbital relaxations via Stochastic-GASSCF, and direct evaluation of properties that can be extracted from density matrices, such as the spin expectation value. Three test case applications have been chosen to demonstrate the flexibility of Stochastic-GAS: (a) the Stochastic-GASSCF [5·(6, 6)] optimization of a stack of five benzene molecules, that shows the applicability of Stochastic-GAS toward fragment-based chemical systems; (b) an uncontracted stochastic MRCISD calculation that correlates 96 electrons and 159 molecular orbitals, and uses a large (32, 34) active space reference wave function for an Fe(II)-porphyrin model system, showing how GAS can be applied to systematically recover dynamic electron correlation, and how in the specific case of the Fe(II)-porphyrin dynamic correlation further differentially stabilizes the ^3^E_*g*_ over the ^5^A_1*g*_ spin state; (c) the study of an Fe_4_S_4_ cluster’s spin-ladder energetics via highly truncated stochastic-GAS [4·(5, 5)] wave functions, where we show how GAS can be applied to understand the competing spin-exchange and charge-transfer correlating mechanisms in stabilizing different spin-states.

## 1 Introduction

Multiconfigurational Self Consistent Field (MCSCF) methods are well-established approaches in quantum chemistry to investigate the electronic structures of systems featuring strong electron correlation effects, and are characterized by highly multireference wave functions. MCSCF wave functions are written as linear combinations of electronic configurations, which can for example be Slater determinants (SDs) or spin-adapted configuration state functions (CSFs). The many-body wave function is then optimized to minimize the CI energy, while the molecular orbitals are self-consistently optimized under the *mean field* of the CI wave function. MCSCF approaches represent a highly flexible strategy that can easily be adapted to a large variety of challenging chemical systems.

The Complete Active Space Self Consistent Field (CASSCF) method, is a popular MCSCF approach.^1−4^ In CASSCF a number of important orbitals, *n*, usually around the frontier orbitals, and their *N* electrons are selected to form the *active space*. Doubly occupied and empty orbitals not included in the active space form the inactive and the virtual spaces, respectively. All possible electronic configurations are generated, compatibly with spin and space symmetry, by distributing the *N* active electrons among the *n* active orbitals, a CAS(*N*, *n*). While the CI coefficients are obtained via exact or approximated schemes for the CI Hamiltonian diagonalization, the orbitals are variationally optimized via inactive–active, active–virtual, and inactive–virtual orbital rotations. CAS is conceptually simple because only one active space has to be selected. However, the size of the CAS wave function exponentially grows with the size of the active space, and the computational costs of conventional diagonalization techniques^5−7^ quickly reach their practical limits for CAS(18, 18) wave functions.

For most chemical systems, the full CI expansion in the active space is unnecessarily large, since CAS wave functions are generally sparse, mostly containing “deadwood”, that is electronic configurations with vanishingly small CI amplitudes.^8−10^ Various methods exist that attempt to exclude deadwood from the CI optimization step, either via a sparse wave function representation, via a user preselection of truncated CI expansions, via an on-the-fly selection of the important electronic configurations, or by exploiting redundancies within the many-body wave function.

One example is the Full Configuration Interaction Quantum Monte Carlo (FCIQMC) algorithm^108−14^ that takes advantage of the sparsity of the wave functions, and deadwood is not (or rarely) processed and stored along the FCIQMC optimization procedure. FCIQMC is a projective method that stochastically propagates the imaginary-time Schrödinger’s equation to solve the CI-problem. Apart from being a sparse CI-eigensolver it can be near-linearly parallelized to benefit from modern hardware. The use of FCIQMC as the CASSCF CI-eigensolver within the Super-CI framework, termed Stochastic-CASSCF,^15^ was developed in our group and has been applied with great success to circumvent the active space size limits of conventional CASSCF.^15−18,97^

Examples of methodologies where truncated CI spaces are preselected include the generalized valence bond approach,^19^ constrained-CASSCF (CCASSCF),^20^ quasi-CASSCF (QCASSCF),^21^ restricted-CI (RCI),^22,23^ restricted active space self-consistent field (RASSCF),^24,25^ the occupation restricted multiple active spaces self-consistent field (ORMAS-SCF) method,^26^ and the generalized active space self-consistent field (GASSCF) approach.^24,27−30^ In CCASSCF the active space is partitioned into several subspaces with a fixed number of particles per subspace; the method is formulated in a basis of CSFs. QCASSCF works like CCASSCF but is formulated in a basis of Slater determinants. RAS wave functions are defined using three active subspaces, commonly labeled RAS1, RAS2, and RAS3, with RAS1 containing doubly occupied orbitals, RAS3 containing empty orbitals, and RAS2 containing orbitals with occupation numbers ranging from 0 to 2. The maximum number of holes in RAS1 and the maximum number of particles in RAS3 are used as restrictions to define the configuration interaction space. In the ORMAS-SCF method, implemented in the GAMESS-US chemistry software package,^31^ several active spaces are chosen; all intraspace excitations are allowed while the number of interspace excitations are restricted by *local* minimum and maximum occupation numbers per active subspace. The corresponding CI problem is solved in the Slater determinant basis, relying on the Slater–Condon rules. The similar concept of generalized active space (GAS) was introduced by Jeppe Olsen already in 1988. In 2011, the GAS approach was coupled to the Super-CI algorithm within the (Open)Molcas chemistry software package^32,33^ for the variational orbital relaxation, leading to GASSCF.^27^ As in ORMAS, the truncated GAS wave functions are built by selecting a number of active subspaces, and imposing constraints at the level of the interspace excitations. However, GASSCF differs from ORMAS-SCF in a number of aspects; most notably, in GAS interspace excitation constraints are enforced via *cumulative* minimum and maximum occupation numbers, instead of the *local* constraints of the ORMAS scheme, and a spin-adapted basis of CSFs is used in the GAS method, relying on the Graphical Unitary Group Approach (GUGA).^34^ GAS-like truncated CI wave functions have also been implemented in the Molpro package.^35^

GAS restrictions can be used to exclude deadwood configurations and to reduce the computational costs while retaining highly accurate multireference predictions. This strategy was adopted in the 2011 work and applied to the dissociation curve of the Gd_2_ dimer and to the study of the relative stability of two energetically low-lying spin states of the Oxo-Mn(salen) complex.^27^ The GAS strategy can also be applied to investigate the role of specific electron correlation mechanisms, by removing electronic configurations that are relevant to describe those correlation pathways. This strategy was undertaken in our group to quantify the effect of the correlation enhanced π-backdonation in Fe(II)-porphyrins,^18^ to understand correlation effects in corner-sharing cuprates, and to investigate the effect of a novel combined approach based on localization, site ordering permutations and GUGA.^36,97^

Selected CI methods are another class of MC techniques that attempt to circumvent the exponential scaling limitation by selecting the important electronic configurations on the fly using automated heuristics. These methodologies heavily rely on the Slater–Condon rules and are generally bound to a Slater determinant basis.^37−45^ Another notable strategy that reduces the exponential scaling limitation is the Density Matrix Renormalization Group (DMRG) theory.^46−56^

In this work we introduce a flexible Stochastic-GAS method, that stochastically optimizes truncated GAS wave functions expanded in the Slater determinant many-body basis, based on the FCIQMC algorithm. In one of our earlier works, we introduced a prototype Stochastic-GAS implementation that supported only disconnected GAS subspaces, in that similar to the QCAS strategy, and successfully applied it to an Fe(II)-porphyrin model system,^18^ to probe the effect of the correlation enhanced σ-donation/π-backdonations on the basis of a large CAS(32, 34) active space.^16^ The GAS algorithm here described also supports interspace excitations that can be restricted by both *cumulative* and *local* minimum and maximum occupation numbers constraints, as in the conventional GASSCF method,^27^ and in ORMAS-SCF,^26^ respectively.

In Stochastic-GAS, occupation number constraints (local or cumulative) are embedded within the precomputed heat bath (PCHB) excitation generation.^57^ Our algorithm does not incur runtime overhead to adhere to the GAS constraints, instead they are automatically accounted for by precalculated heat bath probability distributions. Moreover, the Stochastic-GAS dynamics automatically benefits from another recent development in FCIQMC, the adaptive shift with an offset,^58,59^ that greatly improves the convergence with respect to walker numbers.

Stochastic CAS, QCAS, RAS, and equivalently uncontracted multireference configuration interaction (MRCI) wave functions are special cases of the GAS strategy; thus, they are promptly available by an appropriate choice of the GAS subspaces and corresponding constraints. Our efficient implementation of the Stochastic-GAS method, using hybrid parallelization, the GAS-PCHB excitation generator, and the adaptive shift has allowed, for example, uncontracted stochastic-MRCISD calculations with up to 96 electrons and 159 orbitals and a large (32, 34) active space reference wave function.

Within the Stochastic-GAS method, one- and two-body reduced density matrices (RDMs) can be stochastically sampled as for stochastic FCI or CAS wave functions.^15,60−62^ Those can be subsequently utilized to calculate orbital gradients, Hessians, or within the Super-CI theory^2,27^ to variationally relax the molecular orbitals. This gives rise to Stochastic-GASSCF. As shown in the following, RDMs can also be utilized to calculate properties, such as the spin expectation value. The Stochastic-GAS method has been implemented and has been made available in the open source NECI program.^14^ The Stochastic-GASSCF variant is available via the interface of the NECI code with the OpenMolcas chemistry software package.^33^

The remainder of the article is organized as it follows: In section 2 we summarize the key concepts of GAS and the original PCHB algorithms. In section 3 we introduce the novel GAS-PCHB method, and discuss in some details its performance. In section 4 we discuss three test case applications, that show how Stochastic-GAS can be applied to various chemical situations, and to understand the role of different forms of electron correlation mechanisms. The first example is a stack of five benzene molecules, at varying intermolecular distances, which illustrates the applicability of Stochastic-GASSCF to fragment-based chemical systems. The second example uses Stochastic-GASCI to perform a very large uncontracted stochastic MRCISD calculation that correlates 96 electrons and 159 orbitals, and uses a large CAS(32, 34) active space reference wave function, for an Fe(II)–porphyrin model system, and demonstrates how our algorithm can be used to account for dynamic correlation effects. With this example, we also demonstrate that dynamic correlation effects outside the CAS(32, 34) further stabilize the ^3^E_g_ over the ^5^A_1g_ spin state. In a third test case application, the Stochastic-GASCI strategy has been utilized to investigate the low-energy spin ladder of an Fe_4_S_4_ cubane cluster. We show how the GAS strategy can be applied to understand the two competing spin-exchange and charge-transfer correlating mechanisms in stabilizing different spin-states. In section 5 we summarize the findings of this paper and section 6 contains an appendix with mathematical details.

## 2 Theoretical Background

### 2.1 Generalized Active Space (GAS) Wave Functions

The generalized active space approach arises from the necessity to build truncated CI wave functions that span a preselected portion of the corresponding complete active space (CAS). As for CAS, GAS-CI wave functions are preselected by the user, through chemical (and/or physical) considerations, and a careful choice of active orbitals and electrons. The active orbitals are subsequently partitioned in a number of active subspaces. The nature, size, and number of these subspaces largely depend on the investigated systems, and generally are chosen according to the type of electron correlation that one wants to target or exclude from the CI space. The examples discussed in the Application section or in ref (27) can be used as guidelines to the strategic choice of GAS subspaces.

**Figure 1:**
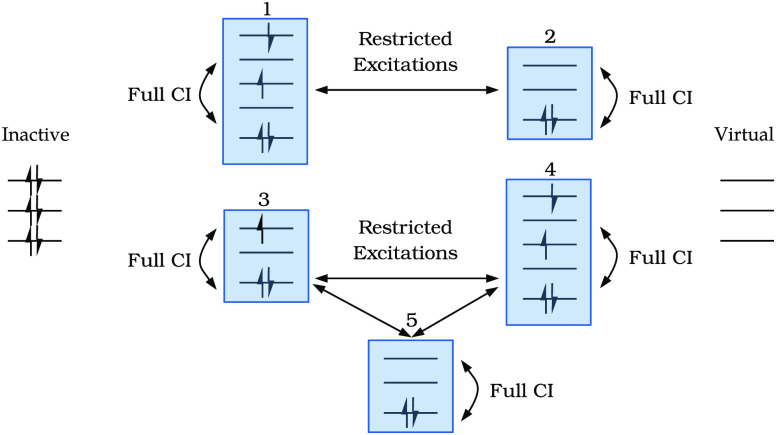
Pictorial representation of a GAS wave function with five GAS subspaces. GAS1 and GAS2 are connected to each other but disconnected from the rest. GAS3, GAS4, and GAS5 are also connected to each other but disconnected from GAS1 and GAS2.

Within each subspace a full-CI expansion is generated (complete set of intraspace excitations), while the number of interspace excitations is restricted.^18,24,27,28^ GAS spaces are defined *disconnected* if no interspace excitations are permitted, while they are defined *connected* if interspace excitations are allowed. In the same GAS wave function both connected and disconnected spaces can exist. Figure 1 depicts a possible specification of GAS constraints.

The number of interspace excitations are limited by constraining the particle number per GAS space. In the original work on the GAS approach^27,63−65^ the term *supergroup* was utilized to refer to a given distribution of particles (α-, β-, or in general electrons) among GAS subspaces, while fulfilling the GAS constraints. We will interpret *supergroups* as a special case of *compositions*, a term borrowed from number theory.^66^ A composition is a solution to the following integer equation
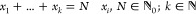
1We consider two *compositions* to be different, if their order differs, that is, 2 + 1 = 3 and 1 + 2 = 3 are two different compositions. If we identify the number of summands *k* with the number of GAS spaces, *N* with the total number of particles, and *x*_*i*_ with the number of particles in the *i*th GAS space, we can easily interpret a given composition as distribution of particles over GAS spaces. We can constrain the allowed compositions, hence the allowed interspace excitations, by defining *local* or *cumulative* minimum and maximum occupation numbers per GAS space. We write *N*_*i*_^min^, *N*_*i*_^max^ for local constraints and *Ñ*_*i*_^min^, *Ñ*_*i*_^max^ for cumulative constraints. GAS allowed compositions are then those for which
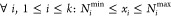
2
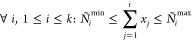
3is fulfilled and will be called *supergroups*, as mentioned before. Two electronic configurations with the same distribution of electrons per GAS subspace belong to the same supergroup.

The occupation number constraints cannot be chosen freely. For example, if the total minimum exceeds the total number of electrons, ∑_*i* = 1_^*k*^*N*_*i*_^min^ > *N* or *Ñ*_*k*_^min^ > *N*, no valid composition (and supergroup) exists. In chemical applications, the Pauli-principle enforces that the number of spin orbitals in a GAS space is larger than or equal to the minimum particle number. These “constraints of the constraints” have been discussed in the literature.^26^

We trivially note that if minima and maxima equal each other in every GAS space, it is not possible to excite a particle from one GAS space to the other, and the spaces are disconnected. We also note that in the case of cumulative constraints it is possible to enforce tight inequalities for the last space *Ñ*_*k*_^min^ = *Ñ*_*k*_^max^ = *N* which tie the total number of particles and the GAS constraints together and allow shortcuts in an algorithm using cumulative constraints.

It is not always possible to convert between the two types of constraints; there are constraints which can be expressed only using local constraints, and vice versa. This aspect has already been discussed in the manuscript introducing the GASSCF method.^27^ An example is given in the Appendix (example 6.5). If the constraints can be converted into each other it is done by the following relationships (Lemma 6.6, see Appendix):
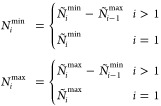
4In the Appendix (subsection 6.2) we give the necessary proofs.

It can be proven easily that CAS and RAS wave functions are special cases of GAS, with one and three active spaces, respectively. We trivially note that in CAS, as only one active space is necessary, a single supergroup is generated. We also note that GAS wave functions with purely disconnected spaces have also one supergroup, with a constant number of electrons per GAS space. On the contrary, the electronic configurations generated in RAS wave functions can already be distributed among a number of supergroups, depending of the level of excitation from RAS1 and into RAS3 spaces.

The conventional GASSCF, that we implemented in 2011 and made available within the Molcas(32) and the OpenMolcas(33) chemistry software packages, is based on cumulative GAS constraints.^27^ The ORMAS-SCF method uses local occupation number constraints.^26^ The novel Stochastic-GAS algorithm introduced in this work allows both local and cumulative constraints.

The MRCI method accounts for dynamic correlation effects on top of a multiconfigurational wave function chosen as reference, generally of CAS type.^67−69^ Since the uncontracted MRCI approach can be expressed via RAS specifications, an efficient GAS algorithm could promptly allow uncontracted MRCI calculations. This is generally prohibitively expensive, considering the unfavorable exponential scaling of RAS wave functions with respect to the size of RAS1 and RAS3 spaces.^32^ However, it is feasible using our Stochastic-GAS algorithm, and an example is offered in section 4.2.

In the GASSCF method orbitals are variationally optimized via a self-consistent field (SCF) procedure, under the mean field generated by the GAS wave function.^27^ As in CASSCF, all intraspace orbital rotations, such as GAS1 ↔ GAS1 or GAS2 ↔ GAS2, are *redundant* and already described by the intraspace excitations in the CI expansion; thus, these excitations are excluded from the orbital optimization. Interspace orbital rotations such as GAS1 ↔ GAS2, however, are only partially redundant and have to be considered in the GASSCF orbital optimization step, in addition to inactive–active, inactive–virtual, and active–virtual rotations. As some of these rotations are already represented by the GAS wave functions, linear dependencies are introduced, that often have a negative impact on the rate of convergence of the GASSCF procedure.^65^

Also of interest is the structure of the 1-RDM for GAS wave functions. For *disconnected* GAS spaces the 1-RDM is block diagonal, because off-diagonal elements, which couple orbitals belonging to different GAS subspaces, vanish. Thus, the diagonalization of the 1-RDM for disconnected GAS, which leads to the natural orbitals, represents an invariant orbital transformation. For connected GAS subspaces, the off-diagonal elements between orbitals belonging to different GAS spaces in general do not vanish, and diagonalization of the one-body density matrix becomes a noninvariant rotation, that mixes orbitals from different GAS subspaces. Thus, natural orbital occupation numbers are only well-defined for disconnected GAS spaces. For connected spaces we can define “pseudonatural orbitals” which are obtained from the block diagonalization of the 1-RDM, each block referring to orbitals of one GAS subspace. Pseudonatural orbitals and natural orbitals are identical for disconnected spaces.

Although GAS wave functions with purely disconnected spaces are highly constrained, they are of great theoretical and practical interest. From a practical standpoint, they do not suffer from the redundancy problems mentioned above, and they have well-defined natural orbital occupation numbers. An algorithm that assumes purely disconnected spaces is also much easier to derive and implement. A first prototype of the stochastic-GASSCF method with disconnected spaces was reported in our earlier work.^18^

### 2.2 Precomputed Heat Bath (PCHB)

In this section, we discuss the Precomputed Heat Bath (PCHB) excitation generation using the Heat Bath sampling algorithm developed by Holmes et al.^57^ in the context of FCIQMC,^57^ and adopted in the present work for the stochastic-GAS algorithm. For the reader who is unfamiliar with FCIQMC we give a brief summary in the Appendix (subsection 6.1).

We first introduce the (on-the-fly) *Heat Bath* excitation generator which calculates matrix elements to all connected determinants on-the-fly and suggests a new determinant with proportional probability. From the generation probability standpoint this is the ideal excitation generator, but the wall clock time per excitation becomes quickly large because an on-the-fly calculation of matrix elements incurs large overhead and the setup of the nonuniform probability distributions scales with the number of orbitals *n* and number of particles *N* as .

The PCHB excitation generator adopted for the Stochastic-GAS algorithm is based on the Slater–Condon rules for double excitations. If we evaluate the matrix element between two determinants that differ only by a double excitation, we obtain
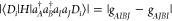
5Thus, the matrix element only depends on the two-electron integrals (*g*) involving the differing orbitals, hence it only depends on the excitation, but not on the starting determinant, *D*_*i*_. This allows the following approximate heat bath excitation generation: starting from a determinant *D*_*i*_, two particles *I*, *J* are selected; next, two indices *A*, *B* are drawn for the holes from a precalculated probability distribution with probability given by
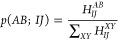
6where *H*_*IJ*_^*AB*^ are the matrix elements for a double excitation from *I*, *J* to *A*, *B*. If the picked hole indices *A*, *B* are already occupied in *D*_*i*_ the excitation is discarded. The numerator of eq 6 involves no approximation. However, compared to the on-the-fly heat bath method, the denominator contains some nonzero elements which would vanish in the nonapproximated heat-bath algorithm if *X* or *Y* were already occupied in *D*_*i*_. Note that *p*(*AB*; *IJ*) is not yet the complete *p*_gen_ value needed for eq 25. It has to be multiplied with the probability to perform a double excitation and to draw the particles *I* and *J*.

If the probability distribution from eq 6 is implemented using the alias-method, the time for the excitation generation scales as  with the number of orbitals and particles.^70^ As we will discuss later in depth, this is a typical trade of “space for time”. If we write |*M*| for the number of elements in a set *M*, we need |{(*I*, *J*)|*I* < *J*; *I*, *J* < *n*}| probability distributions with |{(*A*, *B*)|*A* < *B*; *A*, *B* < *n*}| entries; hence, the memory demand scales with , with *n* being the number of orbitals.

The Hamiltonian matrix element between two determinants *D*_*i*_ and *D*_*j*_ that differ by one single excitation is given by
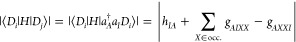
7The value of this element depends on the specific occupied orbitals in *D*_*i*_. Hence it is not possible to define configuration-independent probability distributions as for the double excitation case. Thus, in general it is not efficient to use precomputed probability distributions for single excitations as it cannot be done in  time.

In the context of single-reference methods it is possible to introduce additional approximations and define precomputed probability distributions even for single excitations.^71^ However, for the more general case of multireference wave functions, which represent our main target, such approximations cannot be applied, and single excitations are picked uniformly.

## 3 The GAS-PCHB Algorithm

In this section, we describe how the PCHB excitation generation and the concept of supergroups in GAS can be combined to derive an efficient algorithm, that we call GAS-PCHB, for performing Stochastic-GASCI and Stochastic-GASSCF calculations within the FCIQMC framework.

The simplest stochastic implementation of GAS constraints consists in performing excitations using the conventional FCIQMC excitation generators and to discard GAS forbidden excitations *a posteriori*. The *discarding* GAS implementation can be easily combined with any already available FCIQMC excitation generator, including PCHB, and represents the natural choice for benchmarking more sophisticated GAS excitation generators, such as the GAS-PCHB algorithm, that *a priori* suggests only GAS allowed determinants. We have also implemented a discarding-GAS algorithm and found that when GAS constraints simply aim at removing deadwood configurations, the discarding-GAS performs surprisingly well, and it is rather challenging to develop GAS excitation generators that aim at excluding configurations *a priori*, without incurring overhead that makes the discarding implementation faster in practice. We succeeded in this task via the GAS-PCHB algorithm.

### 3.1 The Algorithm

In FCIQMC the spawning step is responsible for the stochastic propagation of walkers into the CI space, starting from occupied determinants. Thus, if we assume that our starting determinant is allowed by GAS constraints, only the spawning step has to be modified to ensure that all spawned determinants are GAS allowed. The algorithmic details to realize a GAS-PCHB excitation generator are described in this section.

Within the GAS approach, a given (*A*, *B* ← *I*, *J*) excitation can lead to a GAS allowed or forbidden determinant *D*_*j*_ depending on the starting determinant *D*_*i*_. Hence for GAS, it is not possible to generate probability distributions that only depend on the orbital indices, *p*(*AB*; *IJ*) as for eq 6. The concept of supergroups and compositions (section 2.1) are introduced in our GAS-PCHB excitation generator to circumvent the dependency of the probability distributions on the individual Slater determinants.

The supergroup of a given determinant can be determined by counting the particles per GAS space (a  operation, where *N* is the number of correlated particles). Counting how many particles an excitation transfers between GAS spaces is also a trivial operation. Hence for a given supergroup (and all determinants belonging to it), an excitation is GAS allowed if the composition after excitation is still inside the chosen GAS constraints. Whether an excitation is GAS allowed or forbidden only depends on the supergroup of the starting determinant *D*_*i*_. This condition applies for local and cumulative constraints alike.

If we define *i*_sg_ to be a labeling index for the supergroups, we can introduce a modified Hamiltonian, *H̃*(*i*_sg_), for each supergroup whose entries are set to zero for GAS forbidden excitations and to the original Hamiltonian otherwise. Thus, in the case of double excitations, we can write
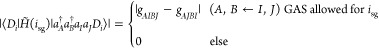
8Equation 8 is similar to eq 5, in that the right-hand side of the equation does not depend on the determinant *D*_*i*_, but only on its supergroup *i*_sg_(*D*_*i*_). Similar to the FCI-PCHB probability distributions (eq 6), we can define GAS-PCHB probability distributions as
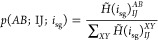
9Although the new dependency on *i*_sg_(*D*_*i*_) increases the number of probability distributions that have to be generated and stored, the direct dependency on the individual Slater determinants is circumvented, making GAS-PCHB a practical tool of general applicability. In the next section the scaling of the algorithm will be discussed together with some examples that show the practical limitations bound to the dependency of *p*(*AB*; *IJ*; *i*_sg_) on the number of supergroups. However, we can anticipate that since there are much fewer supergroups than determinants, the different probability distributions can in most of the practical cases be precomputed and stored.

As for the FCI-PCHB case, it is not possible for single excitations to use precomputed probability distributions to perform importance sampling according to the matrix element. Nevertheless, it is possible to perform uniform selection of holes for single excitations which at least automatically adhere to GAS constraints by using
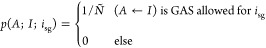
10where *Ñ* is an appropriate normalization factor, to ensure ∑_*X*_*p*(*X*; *I*; *i*_sg_) = 1 for a given particle *I* and given supergroup *i*_sg_. Such a distribution can be very efficiently implemented by using bitmasks.

For an efficient GAS-PCHB excitation generator, a fast function to determine the supergroup index of any given Slater determinant is key. A fast on-the-fly algorithm to calculate *i*_sg_(*D*_*i*_) is given in the appendix (subsection 6.2).

The time to calculate *i*_sg_(*D*_*i*_) can be additionally reduced, by evaluating *i*_sg_(*D*_*i*_) only once for a given determinant and then reusing this value for all walkers on this determinant. The reused supergroup index does not require additional communication, because of an implementation detail in the annihilation step. All walkers on the same determinant are collected to the same process, to facilitate the annihilation of newly spawned walkers from different parent determinants. This implies that in the subsequent spawning step all walkers belonging to a given determinant will reside on one process. Every walker that attempts to spawn from this determinant can look up the index without any communication across processes.

In the special case of disconnected spaces or GAS constraints that are equivalent to CAS there is exactly one supergroup (section 2.1). Hence the index *i*_sg_ equals one for every determinant and does not have to be calculated at all in this case. Algorithm 1 summarizes the main steps of the GAS-PCHB excitation generator.
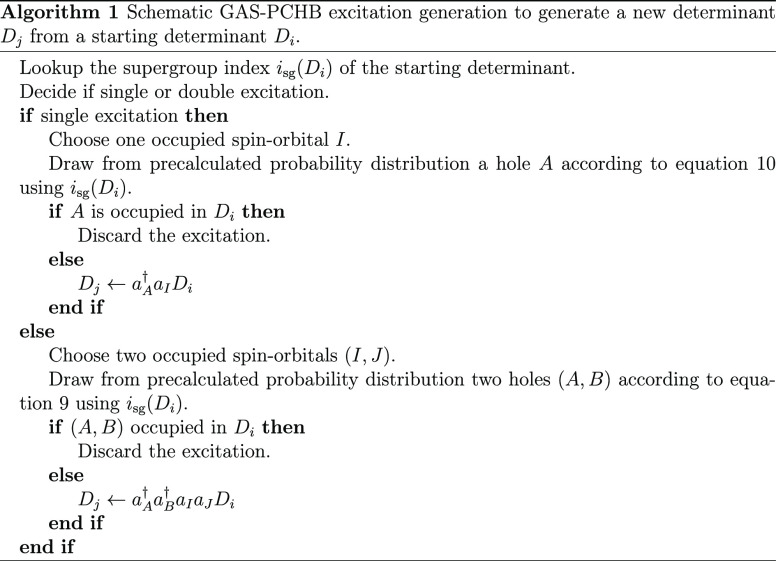


The adaptation of the semistochastic method (introduced in subsection 6.1) to the stochastic GAS-PCHB procedure requires only minor changes, conceptually and code-wise. In the case of GAS, the deterministic core-space Hamiltonian for performing semistochastic FCIQMC dynamics is simply defined as
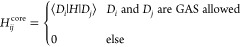
11and since *H*^core^ has to be constructed only once and the full information about GAS constraints is contained in the zeroed off-diagonal elements it is very easy to implement the semistochastic method for GAS constraints.

The sampling of reduced density matrices (RDM) does not require any code adaptation, since GAS forbidden CI coefficients are simply zero and they are not accumulated during the RDM sampling steps.^60−62^ Therefore, the stochastic GAS-PCHB excitation generator allows us to formulate a Stochastic-GASSCF procedure, and gives us access to properties encoded into the RDMs, such as the spin expectation value.

### 3.2 Performance and Scaling

To evaluate the performance of the GAS-PCHB excitation generator, *invariant* and *noninvariant* GAS constraints are to be distinguished. Invariant GAS constraints are those that exclude deadwood configurations. Noninvariant GAS constraints, instead exclude configurations that would have nonzero coefficients in the corresponding CAS-CI expansion, and once removed the resulting total energy increases.

The conventional GAS-CI algorithm,^27^ greatly benefits both from invariant and noninvariant GAS constraints, because the largest bottleneck of the method is the memory required to store the dense CI vector. A truncated Hamiltonian matrix and corresponding CI eigenvector greatly reduce this demand, independently of the nature of the truncated configurations.

Conversely, FCIQMC is a method that benefits from sparsity in the wave function, and unpopulated determinants do not occupy memory and are rarely selected at the spawning step. Hence, invariant GAS constraints do not improve the course of the dynamics, nor do they reduce the corresponding computational costs (spawning process and storage). On the contrary, noninvariant GAS constraints reduce the CI space to which walkers are allowed to propagate. Consequently, these GAS constraints can effectively reduce the computational costs for FCIQMC.

If we compare GAS-PCHB with discarding-GAS and assume that the supergroup index *i*_sg_ is known, and that the list of probability distributions for all supergroups are already available, the drawing of orbital pairs *AB* from *p*(*AB*; *IJ*; *i*_sg_) is practically as fast as drawing *AB* from the corresponding FCI distribution *p*(*AB*; *IJ*). Computational overhead for the GAS-PCHB algorithm arises from the generation of the probability distributions (only at the beginning of the simulation) and from the evaluation of the supergroup index for a given determinant at runtime. In the worst case, if every determinant is occupied by exactly one walker, the supergroup has to be calculated for every walker and the time per excitation increases slightly. Such a dense CI wave function is rarely encountered in practical applications. In actual chemical problems determinants are occupied by multiple walkers and the supergroup index is calculated only once for each newly occupied determinant. Thus, in practical GAS calculations, the evaluation of the supergroup index represents a negligible additional step and the time per excitation can be considered identical for GAS-PCHB and discarded-GAS.

For GAS schemes, where only disconnected spaces are considered, this negligible overhead vanishes completely, since for disconnected GAS schemes only one supergroup exists; the supergroup index *i*_sg_ equals one for every determinant and does not have to be calculated (see section 3.1). This implies that FCI-PCHB can be implemented as a special case of GAS-PCHB.

Since discarded excitations increase autocorrelation of the projected energy, the standard error σ_*E*_ of a discarding-GAS excitation generator will usually be larger than for the *a priori* selection provided by the GAS-PCHB scheme.^72^ Also the *p*_gen_(*i*, *j*) for discarding GAS algorithms is generally lower than for corresponding *a priori* GAS algorithms; this has the effect of leading to a smaller imaginary time-step for the discarding-GAS algorithm. Both effects deteriorate the efficiency of a discarding implementation with respect to the GAS-PCHB algorithm.^57^

Because the amount of GAS discarded excitations strongly depends on the system, it is difficult to give general efficiency ratios between GAS-PCHB and discarding-GAS. But since the time per excitation is in general the same for both methods, GAS-PCHB is usually more efficient than discarding-GAS.

PCHB (in Stochastic-GAS and Stochastic-CAS) is a typical trade of “space for time”. The memory demand for GAS-PCHB probability distributions increases with , where *n* is the number of spatial molecular orbitals and *n*_sg_ the number of supergroups that are generated for a given GAS specification.

It is rather difficult to write a closed expression for the scaling of *n*_sg_ with respect to the number of particles, *N*, GAS spaces, *n*_GAS_, and GAS constraints. It has to be stressed that the number of supergroups is independent from the number of orbitals and in the best case of purely disconnected spaces there is only one supergroup, regardless of *N* and *n*_GAS_. In the worst case of no minimum or maximum restrictions, the scaling of *n*_sg_ is combinatorial and given by the number of compositions (Lemma 6.4) as
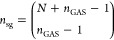
12In practical applications, as the ones discussed in the next section, interspace excitations lie between these extremes, and in general closer to the lower extreme. As an example, we consider a system of five stacked benzene molecules, with an active space that includes the six π-orbitals of each benzene, and distributed into separate GAS subspaces. This system is discussed in greater detail in subsection 4.1. If we use cumulative constraints we can define
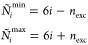
13for the *i*th GAS space, to control the number of allowed supergroups depending on the interspace excitations *n*_exc_. Table 1 shows a steep scaling of the memory with *n*_exc_. Our GAS-PCHB implementation uses hybrid parallelization, and precomputes the probability distributions in shared memory on every node. Thus, in the case of the stack of five benzene molecules, ≈35 GB per node is required for three interspace excitations (Table 1). The memory demand would have been larger with pure message-passing parallelization, where each process requires its own copy of the probability distributions. In that scenario, a 40 processes node would require 1.5 TB of memory for the same system. Since drawing a number is a read-only operation, no complicated locking mechanisms or atomic operations are required, after the distributions have been initialized. The memory demand is further reduced by a factor of  if distribution entries are indexed over spatial orbitals instead of spin orbitals.^14^

**Table 1:** Memory Demand of Probability Distributions for a Hypothetical [5·(6, 6)] GAS Calculation with Different Number of Interspace Excitations *n*_exc_ Using Cumulative Constraints (eq 13)

algorithm	*n*_exc_	*n*_sg_	memory/GB
FCI-PCHB	0	1	0.01
GAS-PCHB	0	1	0.01
GAS-PCHB	1	81	1.17
GAS-PCHB	2	625	9.06
GAS-PCHB	3	2401	34.81

Moreover, as shown in the next section, in practical calculations double interspace excitations are usually enough to recover the Full CI energy for chemically motivated GAS constraints. If the number of interspace excitations is low, the memory demand remains contained and a larger number of particles and GAS subspaces are accessible. For example, in Table 2, we show the memory requirements for a hypothetical [*n*·(6, 6)] GAS calculation, with varying number of GAS subspaces, *n*, and using a fixed number of interspace excitations, *n*_exc_ = 2. On today’s scientific computing hardware, up to eight of such (6, 6) GAS subspaces can be correlated (≈1 TB). In this context, we note again that the number of supergroups is *independent from the number of orbitals*, and only depends on the number of GAS spaces and the level of interspace excitations.

**Table 2:** Memory Demand of Probability Distributions for a Hypothetical [*n*·(6, 6)] GAS Calculation with Varying Number of GAS Subspaces, And a Constant Number of Interspace Excitations Set to *n*_exc_ = 2. The GAS Constraints Are Cumulative as Given by eq 13

*n*_benzene_	*n*_sg_	memory/GB
1	1	0.01
2	5	0.07
3	25	0.36
4	125	1.81
5	625	9.06
6	3125	45.31
7	15625	226.55
8	78125	1132.74

## 4 Application

In this section three applications of Stochastic-GASCI and Stochastic-GASSCF are presented that show how GAS in its stochastic form can be utilized for modeling the electronic structure of a variety of chemical systems. The first example is a stack of five benzene molecules, which illustrates how a limited number of interspace excitations in GASSCF already recovers the full CI energy if the main correlation effects happen inside each GAS space. The second example uses Stochastic-GASCI to perform a very large uncontracted-MRCI calculation for a Fe(II)–porphyrin model system, with a (32, 34) active space as reference wave function, and correlating a total of 96 electrons and 159 orbitals. This example demonstrates how the new method can be efficiently used to account for dynamic correlation in a systematic way. As a last example, we use Stochastic-GASCI to investigate the spin ladder of an all-ferric Fe_4_^(III)^S_4_ cluster, and discuss the role of the leading forms of electron correlation by selectively switching them off via GAS constraints.

### 4.1 Benzene Stack

**Figure 2:**
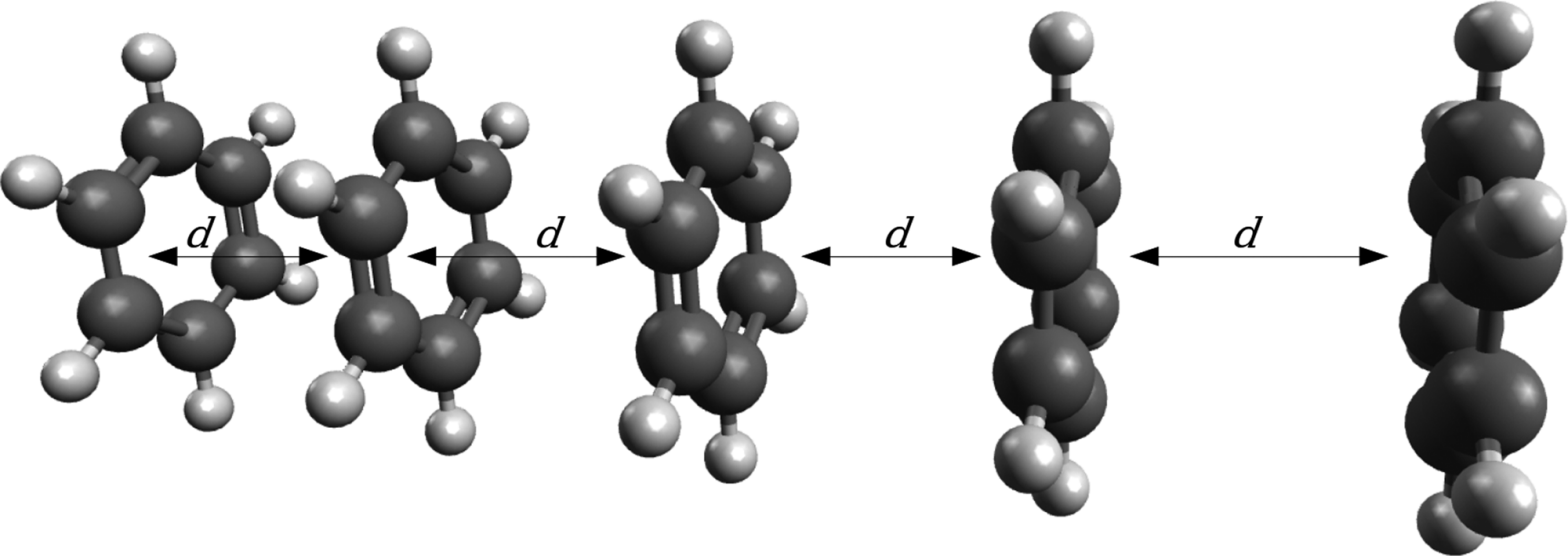
Geometry of the benzene stack. The interfragment distance, *d*, has been changed from a value of 20 Å (very weak, mean-field-only interactions between fragments) to a value of 3 Å, where many-body correlation effects take place.

In this section we discuss the application of Stochastic-GAS-CI and Stochastic-GASSCF to a stack of five benzene molecules separated by a varying distance, *d*, ranging from 3.0 to 20.0 Å (Figure 2).

The geometry of the benzene unit was taken from the Computational Chemistry Comparison and Benchmark DataBase.^73^ A conventional CASSCF(6, 6) calculation was performed on this structure using OpenMolcas.^33^ The resulting MO coefficient matrix was repeated five times along the diagonal to form a block-diagonal coefficient matrix, used as MO basis for the GAS-CI calculations and as starting MOs for the Stochastic-GASSCF optimizations. Since the molecular orbitals of this block-diagonal matrix are not orthonormal, a Gram-Schmidt orthonormalization was performed prior to the stochastic-GASSCF optimization.

For this system, π–π* correlation within each individual benzene (intrafragment) is expected to be dominating, while electron correlation across the fragments is expected to be weaker, and its role becoming increasingly important as the benzene fragments get closer to each other. A GAS [5·(6, 6)] active space has been chosen, which consists of the 30 π-orbitals, six on each benzene, and their 30 electrons. The six π-orbitals of each benzene have been grouped into separate GAS subspaces. We used cumulative
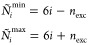
14and local GAS constraints
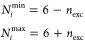
15for the *i*th GAS space. The number of interspace excitations, *n*_exc_, starting from a value of zero (disconnected spaces), was gradually enlarged until convergence in total energy was reached. Local and cumulative constraints are exactly equivalent for disconnected spaces (*n*_exc_ = 0), and yield very similar results for single excitations (*n*_exc_ = 1). Looking at the sizes of the Hilbert spaces, as we will do later in depth, the highest discrepancy between local and cumulative constraints is expected for *n*_exc_ = 1. Since the deviation was negligible already in this case we tested only cumulative constraints for *n*_exc_ > 1.

The case of disconnected spaces, *n*_exc_ = 0, is equivalent to a system of neutral fragments, whose π-electrons are internally correlated and interact among each other only via the mean field generated by the local (6, 6) active space expansion. For *n*_exc_ ≥ 1, charge-transfer configurations are added to the wave function and many-body correlation effects are explicitly accounted for. Hence, convergence with respect to *n*_exc_ was reached earlier for larger distances between neighboring benzene molecules.

The number of supergroups and memory requirements for the different CAS and GAS calculations with cumulative constraints are summarized in Table 1. The CAS(30, 30) space with *S*_*z*_ = 0 consists of 2.41 × 10^16^ SDs, while the GAS space with disconnected spaces consists of 1.32 × 10^14^ SDs which is 0.5% of the CAS size. The connected GAS spaces contain different supergroups for local and cumulative constraints, and the allowed configurations and Hilbert space sizes differ slightly at the same level of interspace excitations. For example, the supergroup [6, 5, 8, 5, 6] would be allowed by the cumulative constraints given in eq 14 for single interspace excitations *n*_exc_ = 1, but is forbidden by the local constraints in eq 15. On the other hand, the supergroup [5, 5, 7, 7, 6] and other multiple single excitations from neighboring fragments would be allowed by local constraints (eq 15) but forbidden by cumulative ones (eq 14).

The [5·(6, 6)] GAS space with *n*_exc_ = 1 and local constraints consists of 51 supergroups and 4.25 × 10^15^ SDs, or 18% of the CAS size, and requires 0.74 GB to store the PCHB probability distribution in memory. Conversely, the cumulative constraints lead to 81 supergroups and 5.22 × 10^15^ SDs, or 22% of the CAS size, and require 1.17 GB of memory to store the corresponding PCHB probability distributions. For a higher number of interspace excitations the difference of Hilbert space sizes between local and cumulative constraints decreases further.

**Figure 3:**
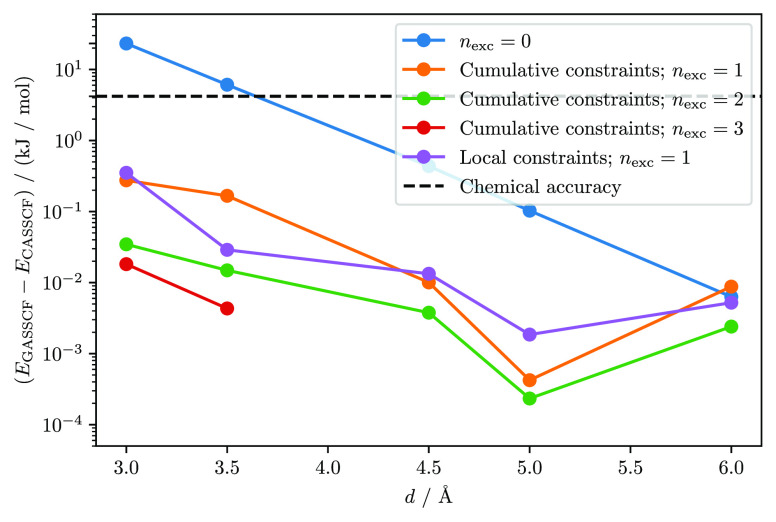
(*E*_GASSCF_ – *E*_CASSCF_) energy difference [kJ/mol] for a number of interspace excitations, *n*_exc_, ranging from 0 to 3, and different distances, *d*, between the benzene fragments. The chemical accuracy of 1 kcal mol^–1^ is marked with the black-dashed horizontal line. A table of all energies is given in the Supporting Information.

Figure 3 shows the energy difference between GASSCF and CASSCF, (*E*_GASSCF_ – *E*_CASSCF_), for a different number of interspace excitations, *n*_exc_, and different distances between neighboring benzene fragments.

The Stochastic-GASSCF energy converges very fast to the Stochastic-CASSCF value, as the *n*_exc_ value is increased. Already with *n*_exc_ = 1 the error is below the chemical accuracy of 1 kcal mol^–1^ for all distances and both local and cumulative GAS constraints.

As expected, the CI-truncation error is dependent on the distance. The closer the benzenes are to each other, the more charge transfer configurations are required for an accurate description of the correlation effects. At an interfragment distance of 4.5 Å and above, disconnected spaces suffice to have deviations smaller than 1 kcal mol^–1^ from the corresponding Stochastic-CASSCF calculation. Notice that this is a typical distance for π-stacking, leading to the conclusion that highly truncated MC wave functions, such as GAS wave functions with disconnected spaces, can be of high value for realistic model systems of weakly interacting fragments. It has to be emphasized that the mean field orbital relaxation effect of fragments onto each other is still accounted for by the SCF procedure, even for disconnected spaces, as opposite to methods where only the CI problem is solved in a fixed MO basis.

The stack of benzene molecules represents a good ground for comparison between Stochastic GASSCF, Active Space Decomposition Density Matrix Renormalization Group (ASD-DMRG),^74,75^ and Non-Orthogonal CI with a Reduced Common Molecular Orbital Basis (NOCI-RCMO),^76,77^ as the latter approaches have also been tested on the same or similar model systems in earlier works. Both NOCI-RCMO and ASD-DMRG are tailored toward clusters of molecules with weak interspace interactions, and share the assumption that the main correlation effects happen within the fragments.

In ASD-DMRG, the CI problem is solved conventionally on each fragment. The compound wave function is then constructed as a linear combination of direct products of fragment states. As in DMRG, a matrix-product ansatz is used for the coefficients.^74^ The dimension of these matrices, commonly called the bond dimension, *M*, is the main factor controlling accuracy and cost of such calculations. Although the *M* value cannot be as intuitively interpreted as the number of interspace excitations in GASSCF, it is also a measure for correlation between fragments. If *M* = 1 the matrix-product reduces to a plain product ansatz of noninteracting systems, while if *M* is the dimension of the full Hilbert-Space, the CI-expansion can be exactly recovered. The *M* value in realistic systems lies somewhere in between, as in those cases *M* cannot be made large enough to reconstruct exactly the entire Hilbert space. The most notable difference between ASD-DMRG and conventional DMRG, is the low value of *M* at the order of 10^2^ that is required by ASD-DMRG to reach convergence for fragment-like systems. Conventional ab initio DMRG, where the sites are not optimized fragments but spatial molecular orbitals requires *M* values that are approximately 2 orders of magnitude higher.

In ref (74), the authors of ASD-DMRG state that “*If a poor initial guess for the chain includes only neutral fragments and the total charge is constrained to be neutral, the algorithm will keep only neutral fragment states although charge transfer configurations may be important in the exact ground state*” and overcome this limitation via a perturtative correction. Within the GAS approach, charge transfer configurations are added by tuning the number of interspace excitations, *n*_exc_. It is thus, possible to precisely identify these configurations and quantify their importance, as shown in Figure 3.

The NOCI-RCMO method uses orthonormal molecular orbitals for each state on each fragment but allows nonorthogonality between orbitals in different states or different fragments.^76,77^ From the nonorthogonal and partly redundant orbitals, a common orbital basis is constructed on each fragment by removing linear dependencies among the orbitals in different states, depending on a cutoff value τ_MO_ for the diagonalized overlap matrix. The common orbital bases on each fragment are then collected together to form a large nonorthogonal MO basis for the cluster. The similarity with the GAS truncation arises at the evaluation of matrix elements which requires several determinant pairs due to the nonorthogonality. Determinants are neglected if their CI-coefficient are smaller than another threshold, τ_det_. The application of NOCI-RCMO to similar aromatic systems as our benzene stack shows that τ_det_ can become as large as 1 × 10^–6^ for fragment distances of 5 Å without affecting the total energy value.^76^ Unlike GASSCF, τ_det_ does not a priori exclude higher-order charge-transfer configurations. However, if a system is made of weakly interacting fragments and fragment MOs are utilized, charge-transfer configurations will have (vanishingly) small CI-coefficients, and will be excluded at run time by the chosen τ_det_ threshold. A high value of τ_det_ has then a similar meaning as a low number of allowed interspace excitations in GASSCF.

It is important to highlight that both ASD-DMRG and NOCI-RCMO are tailored toward systems of weakly interacting fragment molecules, while GASSCF is a method of general applicability, that can be used on compounds of weakly interacting fragments, as well as on strongly correlated and covalently bonded systems as shown in the later sections. Regardless of the chosen method for optimizing the CI problem, the orbital representation is also very important. In the particular case of the benzene stack, choosing fragment-localized orbitals enhances the locality of electron correlation within each fragment, and the sparsity of the many-body eigenvectors. Truncations (via GAS, NOCI, or ASD-DMRG) that take advantage of the sparse structure of the wave functions have negligible impact on the accurate description of correlation effects and on the predicted total and relative energies.

**Figure 4:**
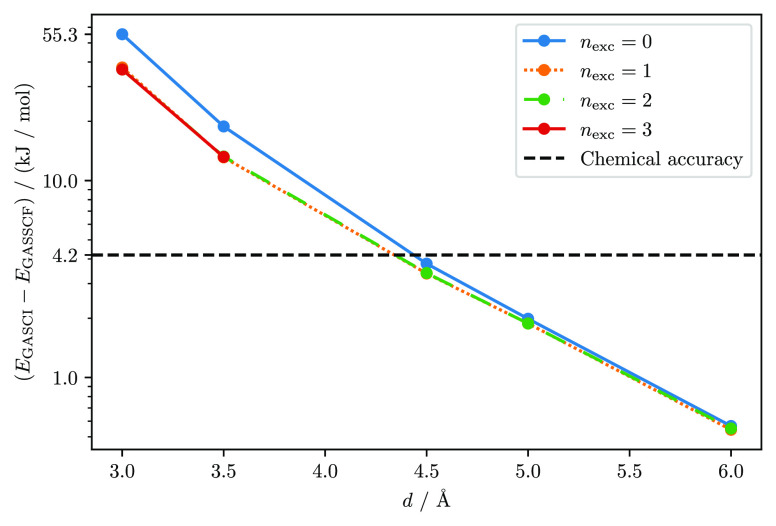
Energy difference between GASSCF and GASCI, (*E*_GASCI_ – *E*_GASSCF_), for different numbers of interspace excitations, *n*_exc_, and different distances between the benzene fragments. The chemical accuracy of 1 kcal mol^–1^ is marked by the horizontal dashed-black line.

In the following, the error introduced by not optimizing the molecular orbitals is discussed. Since the CI energy of the first SCF iteration is the GASCI energy on the initial, unoptimized orbitals, it is possible to compare the GASCI total energies with the GASSCF energies. The energy difference between GASCI and GASSCF (*E*_GASCI_ – *E*_GASSCF_) for different numbers of interspace excitations, *n*_exc_, and different distances between the benzene fragments is shown in Figure 4.

The discrepancy between GASCI and GASSCF, due to missing variational relaxation of the orbitals, is higher than the difference between GASSCF with disconnected spaces and CASSCF, indicating that mean-field effects can be substantially larger than correlation effects bound to the charge-transfer correlation mechanism. Interestingly, the error is nearly independent from the *n*_exc_ chosen.

**Figure 5:**
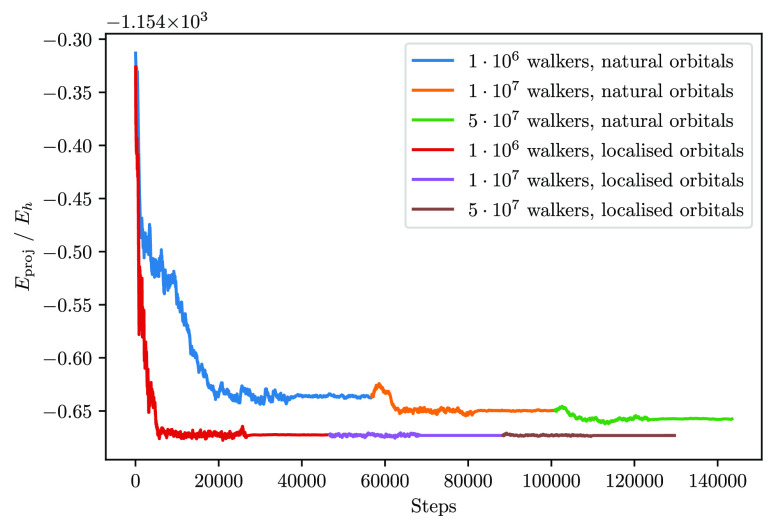
FCIQMC projected energy, *E*_proj_, against the number of iterations using the more delocalized natural orbitals and the fragment-localized orbitals, and increasingly larger number of walkers. The system is the benzene stack (Figure 2) with an interfragment distance of 6.0 Å. The fragment-localized orbitals utilized here are the CASSCF orbitals. The natural orbitals are obtained from the diagonalization of the corresponding one-body RDM.

Of particular interest is also the speed of convergence of the FCIQMC dynamics (in the CAS form) depending on the orbital basis. Only noninvariant orbital rotations are performed in the CASSCF procedure (inactive ↔ active, inactive ↔ virtual and active ↔ virtual rotations). Thus, the active orbitals are not rotated among each other by the SCF procedure, and the main correlation features are to a large extent retained along the CASSCF optimization. At convergence, the optimized CASSCF active orbitals are in general transformed into natural orbitals, by diagonalization of the one-body RDM in the active space. For GASSCF, the diagonalization of the 1-RDM in the full active space is not an invariant rotation. Instead, invariant is the rotation to pseudonatural orbitals, defined as those which diagonalize each GAS subspace separately. While pseudonatural orbitals do not disrupt the fragment-localized structure of the MO basis, the natural orbitals are in general delocalized across the entire system. The locality of correlation (within the fragment) is lost when the more delocalized natual orbitals are utilized, and the many-body wave function generally becomes more dense. FCIQMC dynamics are sensitive to the MO basis adopted. This argument has been discussed for exchange-coupled transition metal clusters.^78^ The benzene stack example shows how the MO representation can affect FCIQMC dynamics for weakly interacting closed-shell systems. The FCIQMC (in CAS form) projected energy, *E*_proj_, as a function of the walker population, and using both natural orbitals and fragment-localized orbitals is depicted in Figure 5.

The FCIQMC dynamics (in CAS form) converge faster when fragment-localized molecular orbitals are utilized, rather than for example the corresponding more delocalized natural orbitals. This result represents the numerical evidence that fragment-localized orbitals produce sparser wave functions which are simpler to describe by a finite distribution of stochastic walkers. Within GASSCF, where the pseudonatural orbitals are used, the convergence speed does not deteriorate, owing to the fact that pseudonatural orbitals preserve the fragment-localized nature of the molecular orbitals. We also note that the time-step can be chosen larger for fragment-localized orbitals () than for delocalized ones () which allows faster propagation along the imaginary time while retaining a stable dynamics. FCIQMC benefits from a sparse representation of the wave function. While the *exact* CI-energy is invariant under unitary MO transformations, methods that approximate the full-CI energy are not invariant under the same MO transformations. This is due to the different degree of sparsity of the Hamiltonian matrix and the corresponding CI wave function with respect to the orbital transformations. If orbitals that are delocalized over the entire compound system are utilized as one-electron basis, a simple π–π* excitation on one fragment can only be represented by a large linear combination of excitations across most (if not all) delocalized MOs, artificially coupling them to each other and, thus, producing unnecessarily complicated CI expansions, featuring long-range entanglements. On the contrary, fragment-localized orbitals keep the leading forms of electron correlation confined within each fragment, increasing the sparsity of the wave function. We also note that for systems made of weakly interacting fragments, such as the benzene stack, and using a GAS strategy that reflects the fragment nature of the compound, the Stochastic-GAS calculations are practically size-extensive if localized orbitals are used, because we can safely assume that only interspace excitations into neighboring fragments are relevant.

### 4.2 Fe(II)–Porphyrin Model System

Iron-porphyrins are the central building block for a variety of enzymes in biochemistry. Owing to the low barrier between Fe^2+^ and Fe^3+^ and nearly degenerate low-energy electronic states, they catalyze important redox reactions and can serve as charge or molecular carriers.^79−82^ The relative stability of the low-energy spin states depends on ligand field and many-body correlation effects experienced by the metal center that, in turn, depend on chemical functionalization and geometry of the conjugated macrocycle. For this reason, a reliable theoretical prediction of the energetically low-lying spin states is challenging and necessary to facilitate the understanding of nature’s efficient enzymatic reactions.

The theoretical prediction of the relative stability of the energetically close ^5^A_1g_ and ^3^E_g_ states in the square planar Fe(II)–porphyrin system is a notoriously difficult task, and there have been a number of theoretical investigations on this topic.^16−18,43,44,83−90^ It has been shown that the triplet is characterized by more complex electron correlation mechanisms than the quintet spin state and only if these correlation effects are precisely taken into account, is the triplet predicted to be the ground state.^16−18^

A (14, 16) active space erroneously predicts a quintet ground state, even when coupled to the post-CASSCF perturbative CASPT2 correction.^16^

If the active space is substantially enlarged, CAS(32, 34),^16^ consisting of the entire ligand-based π system, the σ donating orbitals, and the valence and double-shell d orbitals, complex mechanisms such as 3d–3d′−π* excitations are observed in the wave function^18^ that can be ascribed to correlation-induced delocalization of a metal charge to the macrocycle, a correlated π-backdonation. Only then is a triplet ground state predicted.^16^ In a joint FCIQMC and DMRG study, we have also analyzed the CI-expansion of the wave function and the orbital entanglement to visualize the complex correlation mechanisms taking place in this system.^18^ In the same work, a prototype Stochastic GAS implementation for disconnected spaces was used to quantitatively probe the importance of π-backdonations and was found to have an effect of 2.5 kcal mol^–1^ on the spin gap.^18^

Even if the (32, 34) active space describes qualitatively well the necessary correlation mechanisms, dynamic correlation effects exist that go beyond the (32, 34) active space. Semicore correlation has been accounted for by further enlarging the active space, CAS(40, 38), including the 3s and 3p orbitals, and ultimately increasing the spin gap to 4.4 kcal mol^–1^, at the Stochastic-CASSCF level of theory.^17^ Coupled cluster calculations with up to quadruple excitations (CCSDTQ) inside the Stochastic-CASSCF(40, 38) orbital space yielded a spin gap of 4.8 kcal mol^–1^.^17^ Several methods exist to treat efficiently dynamic correlation effects on top of CASSCF wave functions. However, the list dramatically reduces when a large CASSCF wave function is used as reference. The multiconfiguration pair–density functional theory, MCPDFT, is one of the few methods that can be effectively coupled to very large CAS reference wave functions. MCPDFT calculations on top of DMRG-CASSCF(32, 34) active space were performed by Zhou et al.^89^ and further stabilized the triplet over the quintet with an estimated spin gap of 16.1 kcal mol^–1^.^89^ Although this method can be coupled to large CAS wave functions, it is not possible to systematically improve it. Moreover, although the *delocalization error*(91) does not affect the SCF procedure in MC-PDFT, as this is carried exclusively using the preceding CASSCF procedure, it is possible that the delocalization error bias, dependent on the chosen exchange and correlation translated functional, still exists that overstabilizes the triplet spin-state. Another example is the tailored coupled cluster approach (TCC) that performs Full CI within the active space and uses the resulting CI coefficients as fixed amplitudes in the subsequent coupled cluster equations, which are then solved to account for the remaining dynamic correlation.^92^ The tailored distinguishable cluster method^109^ with singles and doubles (TDCSD) and F12 correction^93,94^ gave a spin gap of 5.8 kcal mol^–1^.^95^

For the current application, the Stochastic-GAS approach has been applied to build and stochastically solve a large RAS-CI wave function. The converged CASSCF(32, 34) MOs have been used as a one-electron basis.^16,18^ The 34 active orbitals have been included in the RAS2 space. The RAS1 space was selected by identifying plateaus in the orbital energy of the inactive orbitals for each irreducible representation and including orbitals above these plateaus. In total 32 doubly occupied orbitals were chosen for the RAS1 space, including the four 3s and 3p semicore orbitals from the metal center and the 28 additional σ-orbitals from the macrocycle. Since there were no well-defined plateaus in the orbital energies of the virtual orbitals, the RAS3 space was simply defined by an energy threshold of 0.85 *E*_h_. The threshold was chosen such that the resulting memory demand could be still fullfilled by the smallest node used for these calculations. All virtual orbitals below this threshold were included into the RAS3 space. A total of 93 empty orbitals were selected for the RAS3 space. Up to double excitations out of RAS1 and into RAS3 were allowed, leading to a total of nine supergroups and a memory requirement of 97.64 GB for the GAS-PCHB probability distributions.

In total, a RAS(96, 2, 2; 32, 34, 93) active space was selected, where the notation RAS(*n*, *l*, *m*; *i*, *j*, *k*) is used, where *n* represents the number of active electrons, *l* is the maximum number of holes allowed in RAS1, and *m* is the maximum number of electrons allowed in RAS3. Active orbitals are labeled by *i*, *j*, *k* and refer to those placed in RAS1, RAS2, and RAS3, respectively. This scheme correlates 96 electrons into 159 orbitals.

The Stochastic-GAS scheme is conceptually equivalent to a stochastic uncontracted Multi-Reference Configuration Interaction approach with single and double excitations from the occupied space (Stochastic-MRCISD). Clearly, no conventional uncontracted or contracted MRCI procedure can be carried that uses the large CAS(32, 34) reference wave function. In that respect the present calculation is unprecedented and it is only possible using our Stochastic-GAS strategy.

The spin gap, *ΔE* = *E*(^5^A_1g_) – *E*(^3^E_g_), predicted by our large Stochastic-GAS approach, is 7.0 kcal mol^–1^, a value that is considerably larger than any systematically improvable result previously reported. The Stochastic-GAS spin gap is reported in Table 3 together with the results obtained with other methods on the same model system. Computational details related to the Fe(II)–porphyrin applications can be found in the Supporting Information.

**Table 3:** Spin Gap *ΔE* = *E*(^3^A_1g_) – *E*(^3^E_g_) between the Quintet and Triplet State of Fe(II)–Porphyrin for Different Methods from the Literature^a^

algorithm	*ΔE*/(kcal mol^–1^)
CASSCF(14, 16)/CASPT2^16^	–0.5
Stochastic-CASSCF(32, 34)^16,18^	3.5
DMRG(*M* = 1 × 10^4^) CASSCF (32, 34)^18^	3.5
Stochastic-CASSCF(40, 38)^17^	4.4
Stochastic-CASSCF(40, 38)/CCSDTQ^17^	4.8
Stochastic-CASSCF(40, 38)/CCSDTQ + F12^17^	5.7
Stochastic-CASSCF(32, 34) + TDCSD^95^	2.6
Stochastic-CASSCF(32, 34) + TDCSD_F12_^95^	5.8
DMRG(*M* = 300) CASSCF(34, 35) + MCPDFT(tPBE)^89^	16.1
	
Stochastic-CASSCF(32, 34) + RASCI(96, 2, 2; 32, 34, 93)	7.0(1)

a The results are sorted by increasing spin gap, except for the MRCISD result in the last row which is from this work.

The spin gap increases from 3.5 kcal mol^–1^ to 7.0 kcal mol^–1^, in going from CASSCF(32, 34) to the large Stochastic-RASCI calculations. The doubled spin gap prediction clearly shows the importance of dynamic correction effects on top of an already large active space, that describes most of the valence correlation mechanisms. This unprecedented result should also be compared to the CASSCF(14, 16)/CASPT2 approach used earlier.^16^ While the CASPT2 also aims at recovering dynamic correlation outside the active space, the chosen active space was too small, and important high-order excitations effects (such as the correlation induced π-backdonation discussed in ref (16 and 18)) were missed by the second order perturbative correction. It is also important to emphasize that our approach can be further systematically improved by increasing the excitation level from RAS1 and to RAS3, a study that goes beyond the scope of the present work.

### 4.3 Fe_4_S_4_ Cubane Spin Structure

In this section, the Stochastic-GAS paradigm is used to investigate correlation effects in spin ladders of exchange-coupled polynuclear transition metal clusters, here exemplified by an all-ferric Fe_4_^(III)^S_4_ cubane complex.

The GAS strategy is first applied to an N_4_ tetrahedron model system as a proof of concept. The smaller N_4_ model is chosen to mimic the weak magnetic interactions across the four magnetic centers of the transition metal cubane. In the all-ferric Fe_4_^(III)^S_4_ cubane, each magnetic center is in a local spin *s*_loc_ = 5/2 with five unpaired electrons, for a total of 20 unpaired electrons. The (20, 20) active space is the smallest that can be chosen to describe spin interactions in this system, which is already too large for conventional multiconfigurational techniques. The N_4_ model is characterized by three unpaired electrons per site, a local spin *s*_loc_ = 3/2, and a total of 12 valence electrons. Conventional CAS(12, 12) calculations are routinely feasible and fast and will be used as a reference for comparisons with GAS(12, 12) calculations, in Slater determinant and spin-adapted bases. Considering that the current implementation of the Stochastic-GAS operates on the basis of Slater determinants (SDs), the N_4_ will also be used to address the question of whether spin-pure solutions can be obtained from our SD-based Stochastic-GAS method.

SDs are not necessarily eigenfunctions of the spin operator *Ŝ*^2^, but they are always eigenfunctions of the spin-projection operator, *Ŝ*_*z*_. Since *Ŝ*^2^ and *Ŝ*_*z*_ commute, a basis of joint eigenfunctions exists. If the respective quantum numbers of *Ŝ*^2^ and *Ŝ*_*z*_ are *s* and *m*_*s*_, for a common eigenfunction of *Ŝ*^2^ and *Ŝ*_*z*_ we know that
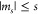
16implying that eigenfunctions of *Ŝ*_*z*_ with eigenvalue *m*_*s*_ cannot form a basis for an eigensolution of *Ŝ*^2^ with |*m*_*s*_| > *s*, but they can form a basis for any eigensolution of *Ŝ*^2^ with |*m*_*s*_| ≤ *s*. Starting from an SD as reference, the FCIQMC dynamics preserves the spin-projection, *m*_*s*_, and converges to the lowest spin state with *s* ≥ |*m*_*s*_|. It follows that for antiferromagnetically coupled systems, it is possible to target spin pure states by adjusting the spin-projection of our starting guess. However, for ferromagnets, where higher spin means lower energy, any spin-unconstrained optimization will inevitably lead to the high-spin ground state, independently of the initial choice of *m*_*s*_. The analysis of the following spin-systems is carried with this limitation in mind.

The distorted N_4_ tetrahedron model system is discussed first. The N atoms are at the equivalent positions of the four metal centers of the Fe_4_S_4_ system. Two N–N bond distances are 2.85 Å, and four are 2.75 Å. The selected active space consists of the 12 2p orbitals. A conventional CASSCF(12, 12) was performed. The optimized natural orbitals were subsequently localized with the Pipek-Mezey method and used as starting orbitals for subsequent GAS-CI calculations. No SCF orbital optimizations were carried for this system, as our main focus is the rationalization of electron correlation mechanisms that are missed with respect to the corresponding CAS, when interspace excitations are sevelery constrained by GAS. This comparison is only possible if the same active orbitals are utilized for CAS and GAS approaches, except for the invariant rotations among the active orbitals.

The GAS active space was defined as [4 × (3, 3)] with the three 2p orbitals of each nitrogen atom grouped in a separate GAS subspace. The charge-transfer excitations between the magnetic centers were controlled using cumulative constraints
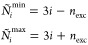
17and excitation levels *n*_exc_ between 0 and 2 were considered. Interestingly, convergence with respect to *n*_exc_ is reached for two interspace excitations, and GAS schemes with larger *n*_exc_ values were not necessary. For *n*_exc_ = 1, the number of supergroups is 19 and a memory of 56.2 MB has been allocated to store the PCHB probability distributions. For *n*_exc_ = 2 the number of supergroups is 85 and 250 MB are required. GAS calculations were performed in both spin pure and SD bases and compared to each other. The Supporting Information contains further computational details.

**Figure 6:**
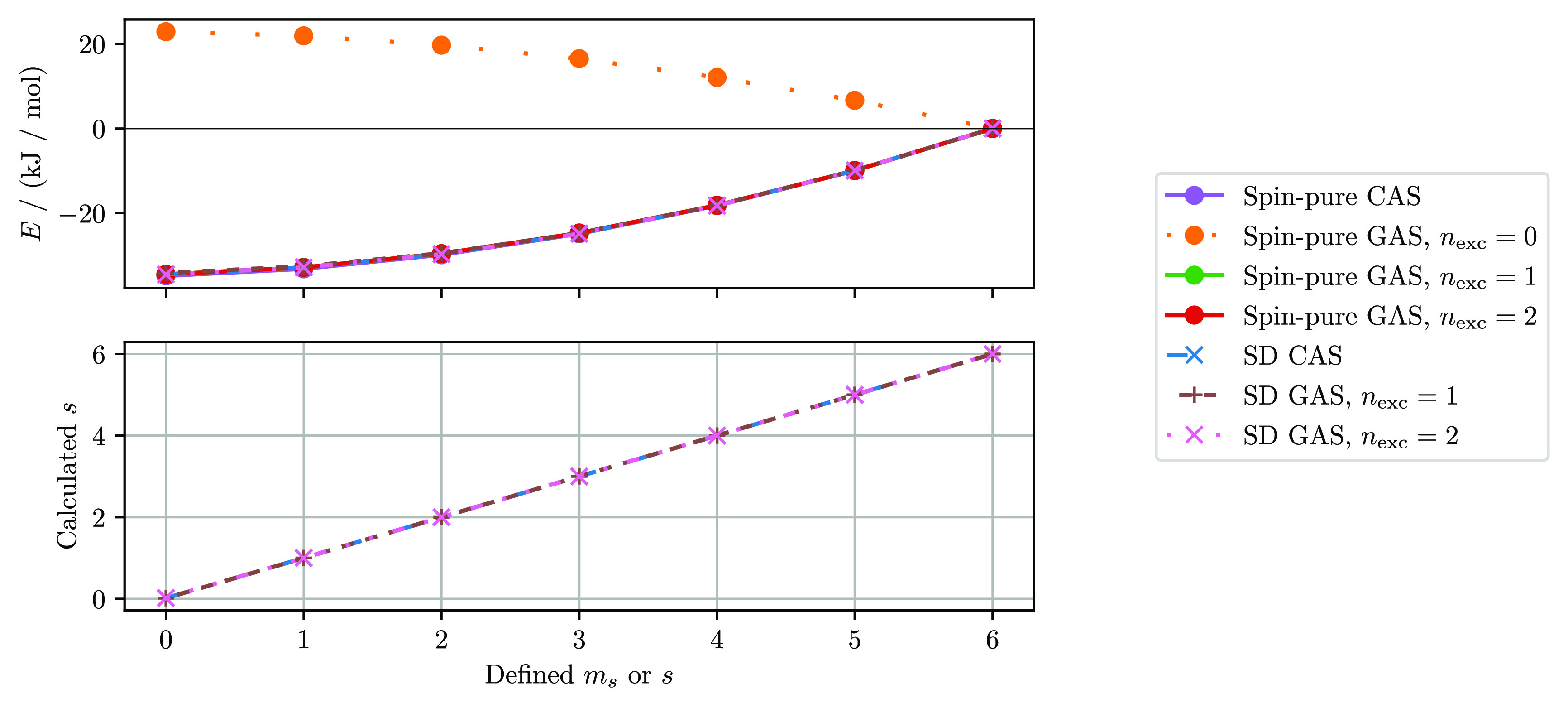
CAS and GAS energies relative to the CAS(12, 12) *s* = 6 state for all lowest spin states of N_4_ and calculated spin quantum number, *s*, for the SD-based CAS and GAS wave functions. Both spin-pure and SD representations were used for GAS. The *x*-axis refers to the total spin, *s*, for spin-adapted calculations and to the total spin-projection, *m*_*s*_, for SD-based calculations. The GAS results in an SD basis for disconnected GAS subspaces are omitted (see main document for details).

The CAS and GAS spin ladders of N_4_, for the different choices of *n*_exc_, and using both spin-pure and SD-based GAS are depicted in Figure 6. In addition, the spin quantum number *s* is computed from the ⟨ψ|*Ŝ*^2^|ψ⟩ expectation value for each chosen *m*_*s*_ value in the SD based calculations and is also reported in Figure 6. In the Supporting Information we derive the working equations for how *Ŝ*^2^ is evaluated using the RDMs.

The highest spin state (*s* = 6) of the N_4_ system can be represented by a single SD (or CSF) with all orbitals occupied by exclusively α (or exclusively β) electrons (|*m*_*s*_| = *s* = 6). The energy of this state is unaffected by GAS constraints, as particles cannot be excited among GAS subspaces anyway, due to the Pauli exclusion principle. For |*m*_*s*_| < 6 numerous forms of electron correlation can potentially take place. Three main excitation types are recognized: (a) on-site excitations (with electron pairing) may lead to non-Hund contributions into the multiconfigurational wave function, (b) exchange interactions, that introduce long-range correlation effects, and (c) charge-transfer excitations across the sites that reduce on-site electron repulsion.

As shown in Figure 6, for spin-adapted and SD-based CAS, the lower spin states of the N_4_ model are energetically more stable than the higher spin states (an antiferromagnet). For GAS wave functions with connected spaces (to the limit of single interspace excitations) the same result is obtained. The agreement between CAS and GAS wave functions with as little as single interspace excitations, *n*_exc_ = 1, is impressive, even though the GAS space is considerably smaller than the CAS space. The CAS(12, 12) space consists of 853776 SDs, while the GAS[4·(3, 3)] space with single interspace excitations *n*_exc_ = 1 consists of 468942 SDs which is 55% of the CAS space size. The largest energy difference between CAS and GAS with single interspace excitations is obtained for the *s* = 0 spin state and is only 0.36 kJ mol^–1^, a negligible quantity.

The N_4_ cluster is antiferromagnetically ordered for CAS and connected GAS spaces, hence we conclude from the previous discussion that we can target spin pure states with selected *m*_*s*_ values and *s* = |*m*_*s*_|. The results in Figure 6 confirm precisely this aspect. CAS and connected GAS energies, obtained using the SD representation, are undistinguishable from the corresponding energies obtained in a spin-adapted basis. Also, the calculated spin quantum number from the expectation value of the *Ŝ*^2^ operator (Figure 6) confirms that for this system all states are pure spin eigensolutions, despite the fact that an SD basis has been utilized.

For disconnected GAS spaces (*n*_exc_ = 0), the spin ladder is inverted to ferromagnetic order. This can be explained by considering the two main competing correlation mechanisms, *spin exchange* and *charge-transfer*. The exchange energy favors parallel alignment of spins across the sites, while charge-transfer correlation across magnetic centers allows for correlation induced differential stabilization of the lower spin states. In the absence of charge-transfer excitations (*n*_exc_ = 0), only exchange interactions remain that stabilize the high spin states, leading to a ferromagnetically ordered system.

Thus, for disconnected spaces, independently of the chosen *m*_*s*_ value for the SD based Stochastic-GAS dynamics, the final state is the one with the highest spin, *s* = 6. For this reason, neither a spin ladder nor spin expectation values have been reported in Figure 6 for disconnected GAS calculations in an SD basis. A spin-adapted Stochastic-GAS implementation is currently under development, that relies on the GUGA technique to build and couple CSFs via the Hamiltonian operator. The development of the spin-adapted Stochastic-GAS is precisely motivated by the above-discussed limitation. It is also important to mention that spin purification techniques exist for ensuring that the eigenvectors of a SD-based CI expansion have the desired ⟨*Ŝ*^2^⟩.^96^

For the Fe_4_S_4_ system, the active space consisted of the 20 3d orbitals of the Fe^3+^ ions and their 20 electrons. The structure of the cluster can be found in the computational details of the Supporting Information. This (20, 20) active space exceeds the limits of conventional CAS; thus, only the stochastic-CAS and GAS strategies will be presented in this section. The spin-pure CASSCF(20, 20) localized orbitals from ref (97) were used, and only CASCI and GASCI calculations were performed here. The GAS active space was defined as [4·(5, 5)] with each of the localized Fe(III) orbitals being in a separate GAS space. While for the N_4_ system, cumulative GAS constraints have been used (for direct comparison with the conventional GAS method where only cumulative constraints are available), the excitation level between the irons was controlled by local constraints
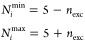
18As shown for the benzene stack, differences between local and cumulative GAS constraints exist, but their practical effect on the energetics is in general negligible. GASCI calculations with an excitation level *n*_exc_ of 1 and 2 were performed. Higher excitation levels were not necessary as convergence is reached already for *n*_exc_ = 2. As in the case of N_4_ it was not possible to use disconnected spaces (*n*_exc_ = 0) due to the ferromagnetic ordering.

**Figure 7:**
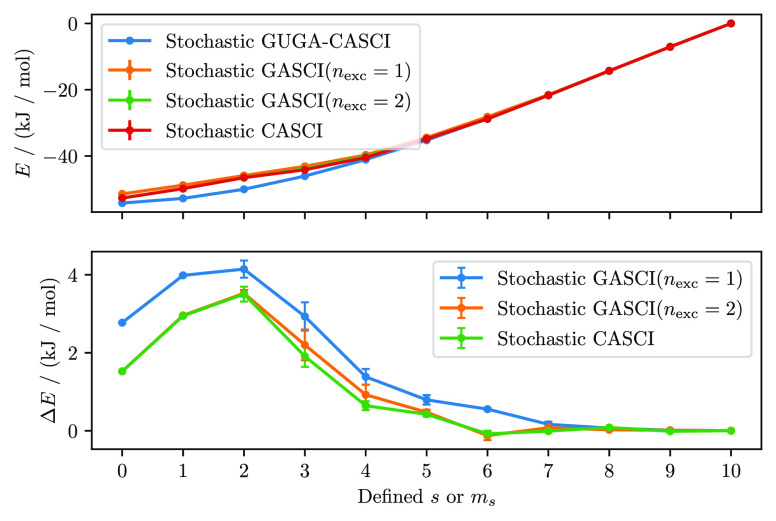
Spin ladder of Fe_4_S_4_ calculated with Stochastic-CAS in SD and spin-adapted basis (GUGA), and Stochastic-GASCI using the SD representation. The Stochastic-CAS and Stochastic-GAS calculations used a defined *m*_*s*_ value to target the desired state, while the spin-adapted Stochastic-CAS used a well-defined *s* value for each state. The upper figure displays the spin ladder energetics relative to the high-spin state (*s* = 10). The lower figure displays the energy difference, Δ*E*, between the SD based calculations and the spin-adapted Stochastic-CASSCF results from ref (97). The local interspace excitations for GAS are controlled with *n*_exc_ according to eq 18. Error bars were obtained using blocking analysis.^72^ The spin-adapted Stochastic-CASSCF calculations used *N*_*w*_ = 1 × 10^6^ walkers,^97^ while the SD-based calculations used *N*_*w*_ = 5 × 10^7^ walkers.

The spin ladder of Fe_4_S_4_ calculated with the SD-based Stochastic-CASCI and Stochastic-GASCI methods is depicted in Figure 7, together with the deviation from the spin-adapted Stochastic-CASSCF results from ref (97).

The analogy between the Fe_4_S_4_ and the N_4_ spin-ladders is to be noted. The highest-spin *s* = |*m*_*s*_| = 10 configuration for Fe_4_S_4_ is an eigenfunction of both *Ŝ*^2^ and *Ŝ*_*z*_, and the CI-expansion consists of precisely one configuration. For this case, neither difference exists between SD and CSF bases, nor between the GAS and the CAS expansions. Moreover, as for the N_4_ system, also the Fe_4_S_4_ system is an antiferromagnet and it is thus possible to target spin eigenstates using the SD representation, without requiring spin-adaptation or spin-purification strategies.

We will first discuss the difference between the SD-based Stochastic-CASCI and the spin-adapted Stochastic-CASCI spin-ladders. Figure 7 shows that the CASCI spin ladder in the SD basis is in good agreement with the spin-adapted CASCI results. Some marginal deviations appear for *s* ≤ 3. The largest deviation occurs for *s* = 2, and it is less than 1 kcal mol^–1^. For higher spin-states the deviation becomes vanishingly small. This difference has been attributed in an earlier work^78^ to the slow convergence of the FCIQMC dynamics in SD basis with respect to the number of walkers (initiator bias). The number of possible SDs for a given number of electrons *N*, orbitals *n*, and spin-projection *m*_*s*_ is given by
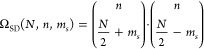
19which reaches its maximum for *m*_*s*_ = 0, if *n* = *N* as in our case. If the initiator bias is directly bound to the size of the Hilbert space, it should be highest for the lowest spin-state. Why, then, is the slowest convergence observed for the s = |*m*_*s*_| = 2 state? The answer is offered by the CI expansion in CSF basis. The Fe_4_S_4_ cluster behaves to the leading terms as a spin-system, with exchange-interactions representing the main form of spin interactions across the sites.^98^ CSFs with singly occupied orbitals represent the leading configurations of such a spin-system, and their number is promptly given by the van Vleck-Sherman formula:^99^
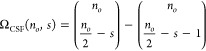
20where *n*_*o*_ and *s* refer to the number of singly occupied orbitals and the target spin, respectively. For the (20, 20) active space, Ω_CSF_ attains its maximum at *s* = 2. The multireference character in a CSF basis is amplified when transformed to the corresponding SD basis, and more walkers are required within the FCIQMC algorithm for a correct stochastic representation of the more complex wave function. Therefore, while it is possible to calculate pure spin states for such systems using an SD basis, the number of walkers required for converging the FCIQMC dynamics is larger than in a CSF basis. While the reference calculation in a spin-pure basis was converged with *N*_*w*_ = 1 × 10^6^ walkers,^97^ the SD-based calculations used *N*_*w*_ = 5 × 10^7^ walkers and was not fully converged in all spin states, as shown by the difference in Figure 7. We would like to stress that the SD-based dynamics were not at the computational limits and increasing the walker number to achieve convergence would have been possible. This was not done as it was not in the scope of the present investigation. The main goal was to compare the truncation effects of GAS compared to CAS.

We now turn our attention toward the error that is introduced from a limited number of GAS interspace excitations. As already observed for the N_4_ system, disconnected GAS model calculations lead to the unphysical inverted and ferro-magnetically ordered spin-ladder. Interestingly, already a GAS with single interspace excitations is enough to obtain a spin ladder in excellent agreement with the corresponding CAS wave function and energetics. The largest deviation from the SD-based Stochastic-CASCI calculation is only 1.2 kJ mol^–1^, and the deviation from the spin-adapted Stochastic-CAS is around 4 kJ mol^–1^ (see lower part of Figure 7). If double excitations among the iron atoms are allowed, the Stochastic-GASCI recovers the CASCI energy with a maximum deviation of 0.3 kJ mol^–1^ from the SD-based Stochastic-CAS energy.

With these examples we demonstrated how the GAS strategy can be used to probe the effect of charge transfer excitations and to reduce the size of the Hilbert space by limiting the number of interspace excitations.

## 5 Conclusion

In this work, the Stochastic-GASSCF method was introduced based on a new PCHB excitation generator in FCIQMC for the stochastic sampling of the GASCI space, the stochastic sampling of RDMs, and the Super-CI method for the variational orbital relaxation. In Stochastic-GAS both local and cumulative particle number constraints can be imposed, a feature that is unique to Stochastic-GAS, and allows exploration of both the cumulative constraints of the conventional GAS,^27^ as implemented in OpenMolcas,^33^ and the local constraints, as implemented in the ORMAS method, and made available in GAMESS-US.^31^ GAS allowed electronic configurations are classified into supergroups based on the number of electrons per GAS subspace. The concept of supergroups is at the core of the new GAS-PCHB excitation generation, since the GAS constraints only require knowledge about the supergroup of a configuration. Since the GAS constraints are ingrained in precalculated probability distributions per supergroup, our algorithm adds practically no runtime overhead to an unconstrained Full CI PCHB calculation. On the other hand, the higher number of probability distributions increases the memory demand compared to Full CI PCHB.

Three different potential showcase applications have been discussed that demonstrate how Stochastic-GAS can be used to reduce computational costs of FCIQMC by operating on preselected truncated CI spaces or to enhance our understanding of the role of different electron correlation pathways.

The first example was a stack of five benzene molecules. We separated the system into five GAS spaces to enable full correlation inside each molecule, but allowed only a limited number of excitations between the fragments. Depending on the distance between the fragments, a different number of interspace excitations was necessary to recover the Full CI energy, but already with one interspace excitation the error was well below 1 kJ mol^–1^ for distances of 3 Å or larger. By this application, we show that Stochastic-GAS can easily be tailored toward fragment-based compounds, and thus operate in a mode that is conceptually similar to other techniques, such as NOCI-RCMO and ASD-DMRG. However, while NOCI-RCMO and ASD-DMRG are exclusively tailored toward fragments, GAS can be applied to a wider range of chemical situations. With suitably chosen orbitals and GAS subspaces the new method is practically size-extensive.

In the second example we used Stochastic-GAS to perform a very large uncontracted stochastic-MRCISD calculation (using the RAS strategy) on an Fe(II)–porphyrin model system, for which a total of 96 electrons and 159 orbitals have been correlated over a (32, 34) RAS2 space. While the RAS2 space accounts for relatively strong forms of electron correlation, as we have discussed already in earlier works,^16−18^ single and double excitations out of the occupied space (RAS1) and into the virtual orbitals (RAS3) account for dynamic correlation. By considering both static and dynamic electron correlation, we could greatly improve the theoretical estimate for the spin gap between the ^5^A_1g_ and ^3^E_g_ state to be around 7.0(1) kcal mol^–1^, substantially larger than our previous estimate exclusively based on the Stochastic-CASSCF(32, 34) energetics. This application shows how Stochastic-GAS can be utilized to account for dynamic correlation in a systematically improvable way, as opposed to other methodologies such as DFT^100,101^ or MC-PDFT^102^ that rely on the accuracy of (translated) functionals to describe exchange and correlation effects.

In the last example, we calculated the spin ladder of an Fe_4_S_4_ cubane cluster and used Stochastic-GAS to understand the role of charge-transfer excitations in differentially stabilizing the low-energy spin-states. We showed that although the current Stochastic-GAS is based on an SD many-body basis, it is possible to efficiently use the method to selectively target pure-spin states of antiferromagnets. A limitation still exists in using the SD-based approach; for some spin states, a relatively slower convergence is observed with respect to the walker number, compared to the spin-adapted implementation. This limitation is independent of the GAS constraints, but depends on the system. This limitation mostly characterizes systems featuring a large number of unpaired electrons, where denser wave functions exist and spin interactions are harder to describe using a SD basis. This limitation is to a large extent removed using spin-adaptation. A spin-adapted Stochastic-GAS strategy is under development and will be presented in a separate work. Via Stochastic-GAS it is demonstrated that the exchange interactions stabilize the high spin states (inverted spin-ladder for disconnected GAS subspaces with ferromagnetically ordered spin states), while low-spin states are stabilized via charge-transfer excitations, that are included as soon as connected-GAS spaces are considered. For the Fe_4_S_4_ cubane system, GAS calculations with single interspace excitations already recover the CAS energy with an error ≤1.2 kJ mol^–1^ and two interspace excitations decrease this error to ≤0.3 kJ mol^–1^.

As a final remark for possible future applications, we note that the Stochastic-GAS strategy can be utilized also for core-excitations, necessary in simulating X-ray diffraction spectroscopy.^103,104^ As for the conventional GAS strategy, core orbitals can be included in one of the GAS subspaces and constrain them to have a minimum number of holes. The advantage of Stochastic-GAS over conventional GAS is that in the former the GAS subspaces can be made substantially larger. Thus, dynamic correlation effects for the core-excited states can be accounted for already at the level of Stochastic-GAS.

Future development of methods in our group will concentrate on GAS in a spin-pure basis using GUGA.

## 6 Appendix

### 6.1 FCIQMC

In this section a brief overview of the FCIQMC algorithm^11,14,105^ is provided, the elements of which are crucial to the understanding of the stochastic-GAS algorithm.

Starting with the imaginary-time (τ = i*t*) Schrödinger equation, , and assuming that an initial state, *D*_0_ (referred to as the reference determinant), has non-zero overlap with the ground state, Ψ_0_, our system will evolve to the ground state in the long-term limit
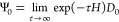
21If we assume a finite many-body basis, for example Slater determinants (SDs), *D*_*i*_, and linearize the propagator via a first-order Taylor expansion, we obtain
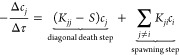
22where
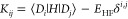
23and *S* is a *shift* parameter specifically introduced within FCIQMC for walker population control. The value of *S* has to equal the correlation energy at stationary conditions.^11^

In principle, eq 22 can be solved deterministically. However, the evaluation of the large number of Hamiltonian matrix elements and the operation count bound to the CI update and the storage of the updated CI vector, makes this equation prohibitive to solve deterministically, in practical cases where large active spaces are utilized. In FCIQMC the imaginary-time evolution of the CI wave function is represented via the propagation of signed stochastic walkers across the configurational space. At each time-step, *Δτ*, the propagation process is divided into four steps: excitation generation, spawning, death, and annihilation. New walkers spawn stochastically using eq 22. For a given time-step *Δτ* we accept new spawns from the parent determinant *D*_*i*_ to the child determinant *D*_*j*_ with an acceptance probability
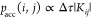
24Note that *p*_acc_(*i*, *j*) may become larger than one which means that a given walker can spawn more than one new walker. For a stable FCIQMC dynamics, it is desirable to have spawn events with a constant probability, hence to keep *Δτ* |*K*_*ij*_| nearly constant. This is achieved by suggesting new determinants *D*_*j*_ with a non-uniform generation probability *p*_gen_(*i*, *j*) ∝ |*K*_*ij*_|, that is, to suggest strongly connected determinants more often. Thus, a modified equation for the acceptance probability is considered
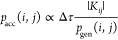
25where a suitable choice of *p*_gen_(*i*, *j*) ∝ |*K*_*ij*_| leads to a more stable acceptance probability. The suggestion of new determinants is called the excitation generation step, and it is at the heart of an efficient implementation of FCIQMC. The excitation generation and spawning steps are “embarassingly parallel” processes. After excitation generation and spawning, the diagonal death step from eq 22 is performed, and all walkers on determinant *D_j_* are stochastically killed with a probability proportional to *(K_jj_– S)*. Depending on the signs of the parent determinants, *D*_*i*_, and of the corresponding *K*_*ij*_ it is possible that spawns to *D*_*j*_ with different signs arise from different determinants *D_i_*, which is a manifestation of the sign-problem within the FCIQMC algorithm.^11,106^ To partially control the sign problem, spawns of opposite sign to the same determinant are summed at each time-step. This process represents the annihilation step.

At the beginning of the simulation the shift parameter, *S*, is kept constant (generally initialized to a small real number or equal to zero). This allows the walker population to grow until the target population is reached. Once the target population is reached, the spawn, annihilation, and death steps are repeated with a shift parameter that is varied such that the target population stays constant. The calculation is carried in stationary conditions to collect sufficient data points for a satisfactory statistical analyis.^72^

The stochastic error of FCIQMC can be greatly reduced by using the semi-stochastic method. After stochastic propagation of eq 22, the *n*_core_ most occupied determinants, representing the *core space* of the evolving CI wave function, are identified, and the full Hamiltonian matrix, *H*^core^, for these configurations is constructed. The dynamics is propagated deterministically inside the core space and stochastically outside.

The projected energy, *E*_proj_ is a common FCIQMC energy estimator which is obtained by projecting the sampled wave function, Ψ(τ), at any imaginary-time, τ, on the reference determinant, *D*_0_:
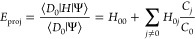
26When the wave function approaches the ground state, the above estimator converges to the ground state energy. To minimize the relative statistical noise of the denominator, one chooses a reference determinant that has a high CI coefficient. This is typically the Hartree-Fock (HF) determinant which usually has the highest CI coefficient. However, if another determinant is found during the simulation to have a higher coefficient, a change of reference may occur, that helps in stabilizing the projected energy estimate. Note that FCIQMC samples of the numerator and the denominator of the projected energy should be averaged separately before taking their ratio.

The original FCIQMC algorithm suffers from a sign-problem in its application to most systems including ab inito ones.^11,106^ When the number of walkers is below a certain threshold, called the annihilation plateau, the sampled wave function does not have a stable sign-structure and is dominated by sign-incoherent noise. The annihilation plateau depends on the system under study and is typically a non-negligible fraction of the overall size of the Hilbert space. This means that one needs a minimum number of walkers that scales exponentially with the number of electrons and the number of orbitals. The problem is largely overcome by applying the initiator approximation, i-FCIQMC, which obviates the annihilation plateau and allows a stable simulation using small numbers of walkers.^12,107^ In i-FCIQMC, a walker is classified as an initiator if the determinant on which it resides has a population above a chosen threshold *n*_add_ (usually set to three). Only initiators are allowed to spawn onto empty determinants, while non-initiators can only spawn onto other occupied determinants. These constrained dynamics stop low-populated determinants from propagating unstable sign-structure further into the Hilbert space but introduce a bias, called the initiator bias, that can be systematically improved by increasing the number of walkers. In the limit of a very large number of walkers, all non-zero walkers become initiators and the exactness of the original method is restored.

The convergence of the initiator method to the exact FCI limit with the number of walkers can be further accelerated with the help of the adaptive shift method.^58,59^ The initiator bias is mainly attributed to the missing back-spawns onto the non-initiators resulting from their underpopulated local Hilbert space. This bias is ameliorated in the adaptive shift method by reducing the shift of non-initiators and thus boosting their lifetime to compensate for the missing back-spawns. In the adaptive shift method, each determinant *D*_*i*_ gets its own local shift *S*_*i*_ as a fraction of the total global shift *S*
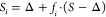
27where *S* ≤ Δ ≤ 0 is an adjustable offset parameter to be discussed below and *f*_*i*_ are factors measuring how much a determinant is affected by the undersampling. These factors are computed during the simulation as a weighted ratio of the spawns accepted under the initiator constraint
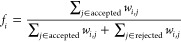
28and the weights *w*_*i*,*j*_ are the first-order perturbation theory contribution of a walker on determinant *D*_*i*_ to determinant *D*_*j*_
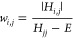
29with *E* being an estimate of the ground state energy such as the instantaneous projected energy.

**Figure 8:**
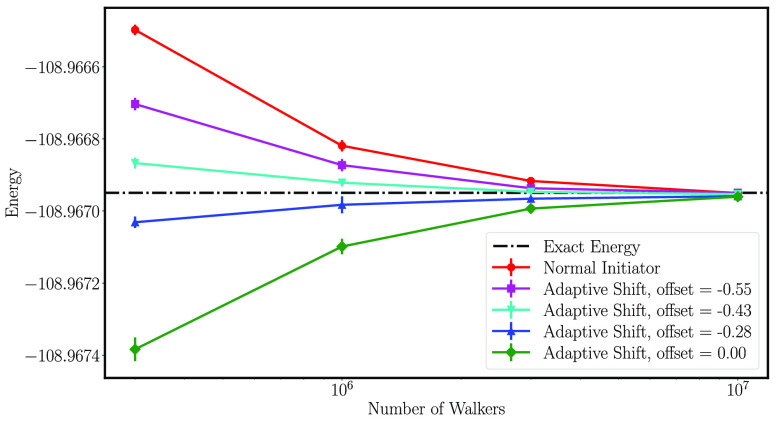
Normal initiator and adaptive shift results using different offsets for N_2_ in the cc-pVDZ basis set and stretched geometry: 4.2*a*_0_.^59^

The offset parameter Δ provides a mean of controlling the amount of correction applied by the adaptive shift method. When Δ = 0, the correction is applied in its full strength (*S*_*i*_ = *f*_*i*_ · *S*), while for Δ = *S* the adaptive shift reduces to the conventional i-FCIQMC algorithm. Lowering the offset gives higher total energy estimates, using the same number of walkers. When the energy is plotted as a function of the number of walkers, there is a strict ordering between the energy curves for different offsets, with some converging from below (high offsets), while others converge from above (low offsets). By varying the offset, one can use this property to bracket the exact energy between the curves of different offsets (see Figure 8). A good starting point for varying the offset is setting it to half the correlation energy Δ = *Ẽ*_correlation_/2. This estimate of the correlation energy, *Ẽ*_correlation_, can be approximated by the shift, *S*, of an earlier FCIQMC calculation or by other methods such as MP2 or coupled cluster calculations.

For the test case applications investigated in this work the choice of Δ = *S*/2 was already satisfactory for largely curing the initiator bias.

### 6.2 Compositions, Supergroups, and Indexing

In this subsection we derive a fast on-the-fly algorithm for calculating the supergroup index of a given determinant. We will first discuss how to calculate the index for a given composition and apply this knowledge to supergroups. We repeat the definitions formally.

**Definition 6.1** (Compositions and Supergroups) For  we call a solution of the following equation
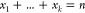
30a composition of integers. We define *p*(*k*, *n*) to be the number of different compositions. Solutions with a different order of summands are considered to be different.

If we identify *n* with the number of particles and *k* with the number of possible GAS spaces then a supergroup is a *composition* that is allowed by local or cumulative GAS constraints.

We define *p*_*l*_(*k*, *n*, *N*^min^, *N*^max^) and *p*_*c*_(*k*, *n*, *Ñ*^min^, *Ñ*^max^) to be the number of supergroups for local and cumulative constraints, respectively.

We trivially note that *p*(1, *n*) = *p*(*k*, 0) = 1 and in general we have the following lemma.

**Lemma 6.2** (Number of compositions) For  the number of compositions is given by
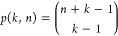
31*Proof.* If there are *k* summands, there are (*k* – 1) “+” symbols in the composition. If we have *n* symbols “∗” and denote numbers with a corresponding number of those symbols we can write compositions with arrangements of “+” and “∗” symbols. For example 8 = 3 + 0 + 3 + 2 can be denoted as 8 = ***++***+**. The number of compositions is then the number of different arrangements.□

**Definition 6.3** (Composition and supergroup index). We assume lexicographical decreasing order of the compositions and assign the composition index based on this order.

In the same way, we assume lexicographical decreasing order of the supergroups and assign the supergroup index based on this order.

In Table 4, we show an example for the composition of three with three summands and the supergroups from example GAS constraints and the respective indices.

**Table 4:** Example for the Supergroups of a GAS Constraint with a Cumulative Minimum and Maximum of [0, 1, 3] and [2, 2, 3]^a^

*i*_sg_	*i*_C_	*x*_1_	*x*_2_	*x*_3_
	1	3	0	0
	2	2	1	0
**1**	**3**	**2**	**0**	**1**
	4	1	2	0
**2**	**5**	**1**	**1**	**1**
**3**	**6**	**1**	**0**	**2**
	7	0	3	0
**4**	**8**	**0**	**2**	**1**
**5**	**9**	**0**	**1**	**2**
	10	0	0	3

a The composition index is *i*_C_ and the supergroup index *i*_sg_. GAS allowed compositions, i.e., supergroups, are in bold font.

**Lemma 6.4** (Determine composition index) For a composition  the composition index *i*_C_ is given by
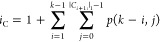
32where
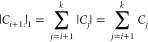
33*Proof.* We know that all elements of the composition lie between 0 and *n*.

If the first element *C*_1_ is equal to *n*, all other elements of the composition have to be zero and the composition index has to be one. (First row of Table 4)

If *C*_1_ is smaller than *n*, all compositions with a leading term *L*, *C*_1_ + 1 ≤ *L* ≤ *n* have a lower index. The number of all compositions with a first element of *L* is given by *p*(*k* – 1, *n* – *L*), because we write the sum of *n* – *L* with *k* – 1 summands.

By summing the number of all compositions with a leading term *L*, *C*_1_ + 1 ≤ *L* ≤ *n*, we can now calculate the index of the first composition which has a leading term of *C*_1_ as
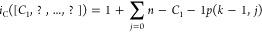
34If we keep in mind that *n* – *C*_1_ = ∑_*i* = 2_^*k*^*C*_*i*_ = |*C*_2:_|_1_ we can rewrite eq 34 as
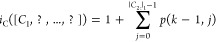
35

If we look at the second element *C*_2_, we are either finished, because *C*_1_ + *C*_2_ equals *n*, or all compositions with a second element *L*, *C*_2_ + 1 ≤ *L* ≤ *n* – *C*_1_ are larger than *C* in lexicographical order. We can repeat the previous steps to calculate the number of compositions and continue this procedure for all elements of *C* to arrive at the final eq 32.□

We give an illustrative example and determine the index of the composition [1, 0, 2]. Since the leading term is a 1, we can “jump” over all compositions with a leading 3 or 2 and arrive at [1, 2, 0], which is the first composition with a leading 1. The number of terms with a leading 3 is given by *p*(2, 3–3) = *p*(2, 0). The number of terms with a leading 2 is given by *p*(2, 3–2) = *p*(2, 1). Then we can repeat the same logic to jump over [1, 1, 1] to arrive at [1, 0, 2]. In total this gives
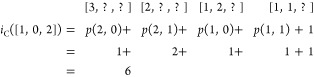
36which agrees with Table 4. Since *p*(*k*, *n*) only requires the binomial coefficient which can be implemented as lookup table, the whole calculation may become a summation of values from a lookup table.

If we had a general closed solution to calculate the number of supergroups *p*_*c*_ and *p*_*l*_, we could apply the same logic of “jumping over” leading terms to calculate the supergroup index. Unfortunately only recursive solutions are known for *p*_*c*_ and *p*_*l*_ which do not lend themselves to an efficient implementation.^66^

For this reason, we generate all possible supergroups in the beginning. This step does not have to be performant, one can, for example, generate all compositions and just test if they adhere to GAS constraints. After lexicographical sorting of the supergroups we calculate the *composition index* for each of them and store it.

If we now want to calculate the supergroup index for a determinant, we count the number of particles per GAS space to get a supergroup, calculate the composition index of the supergroup using Lemma 6.4, and look up the position of this composition index in our stored list. We can calculate for example the supergroup index of [1, 0, 2] as
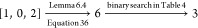
37

### 6.3 Conversion of Constraints

In this section, we want to show that cumulative and local GAS constraints are not always equivalent and that there are systems which can be expressed in only one of them. We also want to prove eq 4 for the conversion between these constraints when it is possible.

We write again *N* and *n*_GAS_ for the number of particles and GAS spaces. If *N*^min^, *N*^max^ denote local GAS constraints and *Ñ*^min^, *Ñ*^max^ denote cumulative ones, then we write for the set of all supergroups under local constraints  and for the set of all supergroups under cumulative constraints .

We start with an example that shows that there is at least one cumulative constraint that cannot be expressed with local ones.

**Example 6.5.** We define a system comprising four GAS spaces using cumulative constraints and eight particles and tight inequalities *Ñ*_4_^min^ = *Ñ*_4_^max^ = 8 in the last space in Table 5. We assume that local constraints *N*^min^, *N*^max^ exist such that 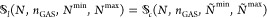
and lead this to a contradiction.

**Table 5:** An Example System with Cumulative Constraints *Ñ*^min^, *Ñ*^max^ which Cannot Be Expressed Using Local Constraints

*Ñ*_*i*_^min^	*Ñ*_*i*_^max^
1	3
4	4
5	7
8	8

*Proof.* The compositions [1, 3, 1, 3] and [3, 1, 3, 1] are contained in . This implies that for all spaces *N*_*i*_^min^ ≤ 1 and 3 ≤ *N*_*i*_^max^. We immediately conclude
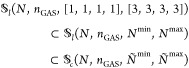
38Since 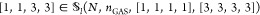
we see from eq 38 that 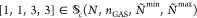
But the calculation shows that [1, 1, 3, 3] cannot be contained in the cumulative GAS constraints from Table 5.□

In a next step, we prove eq 4 with the following Lemma.

**Lemma 6.6.** If for a given number of particles *N* and GAS spaces *n*_GAS_ local GAS constraints *N*^min^, *N*^max^ and cumulative GAS constraints *Ñ*^min^, *Ñ*^max^ exist such that
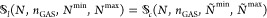
39and if for each *i* > 1 there exists a composition  such that
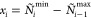
40and if for each *i* > 1 there exists a composition  such that
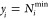
41and if for each *i* > 1 there exists a composition  such that
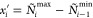
42and if for each *i* > 1 there exists a composition  such that
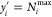
43then the following relationships must hold:
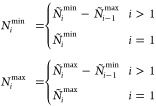
44*Proof.* The relationship is true for *i* = 1. We now prove for *i* > 1 that *N*_*i*_^min^ = *Ñ*_*i*_^min^ – *Ñ*_*i* – 1_^max^. Note that this is not a proof by induction, we just separated the two cases. By definition of *N*_*i*_^min^, we have for the composition from condition 40 that
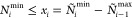
45On the other hand, we have for every composition 
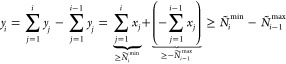
46Since we assume that *y* is also contained in the set of supergroups under local constraints (condition 39), we conclude for every 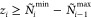
47Since we also assume, that at least one *z*_*i*_ admits the extremal value *N*_*i*_^min^ (condition 41) we conclude
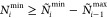
48With inequality 45 we arrive at *N*_*i*_^min^ = *Ñ*_*i*_^min^ – *Ñ*_*i* – 1_^max^. The proof of *N*_*i*_^max^ = *Ñ*_*i*_^max^ – *Ñ*_*i* – 1_^min^ can be performed in exactly the same way.□

It might seem very difficult to use Lemma 6.6 in practice, because the equality of local and cumulative constraints is an assumed condition that has to be verified (eq 39). But it is very useful not so much to convert between constraints that are known to be equivalent, but to prove that a given type of constraint has no equivalent.

We could have proven example 6.5 by directly applying the conversion formulas (eq 4) to the cumulative constraints *Ñ*^min^, *Ñ*^max^. The obtained local constraints *N*^min^, *N*^max^ and the original cumulative constraints fulfill the conditions 39–43, but as in the original proof of example 6.5 the obtained local constraints have different supergroups 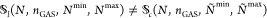
With Lemma 6.6 we conclude that there are no local constraints that are equivalent to the cumulative constraints from Table 5.

## Supporting Information Available

Computational details and the other available files that are necessary to reproduce our results (PDF)
Tables of all results and a derivation of the working equation for the evaluation of the *Ŝ*^2^ expectation value (ZIP)
