# Supplementary material for: Stochastic Generalized Active Space Self-Consistent Field: Theory and Application
Source: J Chem Theory Comput. 2021 Dec 13;18(1):251–72. doi: 10.1021/acs.jctc.1c00936 (PMC8757470; doi:10.1021/acs.jctc.1c00936)
Supplement: Supplementary file 1 — ct1c00936_si_001.pdf [file ct1c00936_si_001.pdf]

Stochastic Generalized Active Space Self-Consistent Field: Theory and Application

Oskar Weser Kai Guther Khaldoon Ghanem
Giovanni Li Manni

1 Computational Details

1.1 Benzene stack

Generally contracted atomic natural orbitals (ANO-RCC) of split-valence, double-ζ plus polarization quality were used (ANO-RCC-VDZP)[1, 2], resulting in a total of 570 basis functions. Scalar relativistic effects were included with the Douglas-Kroll-Hess integral correction. Evaluation of electron repulsion integrals has been accelerated by using the resolution-of-identity Cholesky decomposition with a threshold of $1 \cdot 10^{-3}\,E_\mathrm{h}$.[3–7] For the SCF procedure, a convergence threshold of $1 \cdot 10^{-4}\,E_\mathrm{h}$ over the energy was utilized. The Stochastic-GAS calculations were performed using our new GAS-PCHB algorithm, while the full CAS calculations were performed using the conventional i-FCIQMC algorithm with PCHB excitation generator. A maximum walker number of $2 \cdot 10^8$ has been utilized. The semi-stochastic space consisted of $1 \cdot 10^4$ configurations. The adaptive shift method was employed with a offset value $\Delta = S/2$, where S is the shift value. RDMs were sampled using the non-variational initiator approximation expression:

$$\bar{d}_{PQRS} = \sum_{D_\mathrm{init} \in I} \frac{\langle D_\mathrm{init} | a^\dagger_P a^\dagger_R a_S a_Q | \Psi_\mathrm{total} \rangle}{\langle D_\mathrm{init} | \Psi_\mathrm{total} \rangle} \tag{1.1}$$

as already reported in reference [8]. The energies reported in this manuscript were calculated from these RDMs.

Coordinates The cartesian coordinates for a distance of 4.5 Å can be found in `benzene_4.5_Ang.xyz`.

Molcas input The `OpenMolcas` input to prepare the electronic integrals and to perform the orbital SCF optimization is give in `benzene_4.5_Ang.inp`.

NECI input The `NECI` input for solving the CI problem with FCIQMC is given in `benzene_4.5_Ang.FciInp`. To vary the interspace excitations the arguments to the `GAS-SPEC` keyword have to be adjusted.

Starting orbitals The starting orbitals are supplied in the `OpenMolcas` `RasOrb` fileformat. The file is called `benzene_stack.InpOrb`. These orbitals come from five times repeating the MO coefficient matrix of a (6, 6) CASSCF calculation on a single benzene. The orbitals are *not orthonormal.* The given Molcas input orthonormalizes the starting orbitals depending on the distance of fragments and the resulting AO-overlap.

1.2 MRCI on Fe-porphyrin

All calculations were performed on the same $Fe^{(II)}$-porphyrin model complex as in our earlier work.[8, 9] The pyrrole β-carbon atoms were removed and remaining bonds were capped with H-atoms. The Fe−N bond length was kept at 1.989 Å which is closer to the triplet's equilibrium geometry. The molecule was placed into the xy plane with ligand N-atoms on the x and y axes. The point group of the planar molecule is D_{4h}, however, calculations were carried out using the D_{2h} Abelian point group, the largest our programs can handle.

Basis set and integral specifications have been chosen as in our earlier work [9]. Generally contracted atomic natural orbitals (ANO-RCC) of split-valence triple-ζ plus polarization quality, were utilized (ANO-RCC-VTZP)[1, 2], resulting in a total of 707 basis functions. Scalar relativistic effects were included with the Douglas-Kroll-Hess integral correction. Evaluation of electron repulsion integrals has been accelerated by using the resolution-of-identity Cholesky decomposition with a threshold of $1 \cdot 10^{-3}\, E_\mathrm{h}$.[3–7]

The FCIQMC RASCI-optimization was performed using our new GAS-PCHB excitation generator with initiator approximation and a maximum walker number of $1 \cdot 10^9$. The semi-stochastic space consisted of $1 \cdot 10^7$ configurations. The adaptive shift method was applied with an offset of half the correlation energy. The projected energy is reported for the energy results.

Due to the large scale of this calculation we also want to report the computational cost of the FCIQMC dynamics with $1 \cdot 10^9$ walkers. They were performed on the Max-Planck Computing and Data Facility (MPCDF) HPC system Cobra using 256 nodes, each having two Intel Xeon Gold 6148 (20 cores, 2.4 GHz) processors and 192 GB DDR4 RAM, with the nodes connected through Intel OmniPath interconnect. The calculations used 38 processes and 170 GB of memory per node and ran for $5 \cdot 23$ h resulting in a total of $1.1 \cdot 10^6$ core hours. Since `NECI` is near-linearly parallelized[10], the dynamics with fewer walkers were performed on in-house clusters.

Coordinates The cartesian coordinates can be found in `FePor.xyz`.

Molcas input The `OpenMolcas` inputs to prepare the electronic integrals and to perform the orbital SCF optimizations for the two spin states can be found in `quint.inp` and `tripl.inp`.

NECI input The NECI inputs for solving the CI problem with FCIQMC for the two spin states can be found in `quint.FciInp` and `tripl.FciInp`.

Starting orbitals The (32, 34) CASSCF converged starting orbitals are supplied in the `OpenMolcas RasOrb` fileformat. The files are called `quint.InpOrb` and `tripl.InpOrb` for the two respective spin states.

1.3 Spin exchange in Fe_4S_4

N_4 The basis set consisted of generally contracted minimal atomic natural orbitals (ANO-RCC-MB), resulting in a total of 20 basis functions.[1, 2] The calculations were performed with conventional CI in a spin pure basis using `OpenMolcas` and with FCIQMC in an SD basis using `NECI`.[10, 11]

The FCIQMC CI-optimization was performed using our new GAS-PCHB and the existing FCI PCHB excitation generator with initiator approximation and a maximum walker number of $1 \cdot 10^5$. The semi-stochastic space consisted of $1 \cdot 10^5$ configurations. The projected energy is reported for the energy results.

Fe_4S_4 The cluster is depicted in Figure 1.

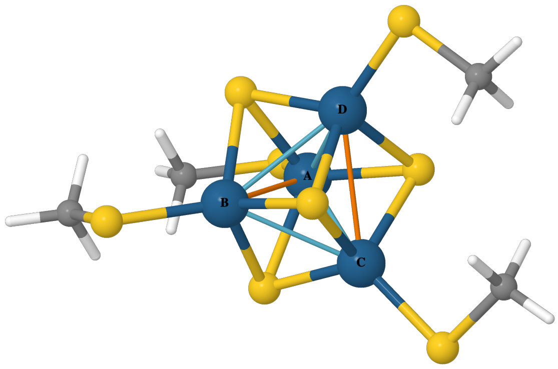

Figure 1: Structure of the Fe_4S_4 cluster.

The basis set consisted of generally contracted minimal atomic natural orbitals on all atoms except iron, where split-valence, double-ζ plus polarization quality functions were used (ANO-RCC-MB, Fe.ANO-RCC-VDZP)[1, 2], resulting in a total of 212 basis functions.

The FCIQMC CI-optimization was performed using our new GAS-PCHB and the existing FCI PCHB excitation generator with initiator approximation and a maximum walker number of $5 \cdot 10^7$. The semi-stochastic space consisted of $1 \cdot 10^4$ configurations. The projected energy is reported for the energy results.

Coordinates The coordinates are saved in `Fe4S4.xyz`.

Molcas input The `OpenMolcas` input to prepare the electronic integrals and to perform the orbital SCF optimization for the $s = 0$ state can be found in `Fe4S4.inp`. The input has to be adjusted for the other spin states. The `spin` argument changes the spin and the `FileOrb` argument changes the file for the input orbitals.

NECI input The `NECI` input for solving the CI problem with FCIQMC is given in `Fe4S4.FciInp`. To vary the interspace excitations the arguments to the `GAS-SPEC` keyword have to be adjusted.

Starting orbitals The CASSCF optimized starting orbitals are supplied in the `OpenMolcas RasOrb` fileformat. The files are called `Fe4S4_S_${s}.InpOrb` where `${s}` goes from 0 to 10 and denotes the spin state.

2 Results

Table 1: Results for the stack of benzene.

d/Å	n_{exc}	E/E_{h}	SCF	constraints
3.0	0	−1154.504 927	T	cumulative
3.0	1	−1154.513 659	T	cumulative
3.0	1	−1154.513 630	T	local
3.0	2	−1154.513 777	T	cumulative
3.0	3	−1154.513 771	T	cumulative
3.0	CAS	−1154.513 764	T	cumulative
3.5	0	−1154.631 333	T	cumulative
3.5	1	−1154.633 592	T	cumulative
3.5	1	−1154.633 644	T	local
3.5	2	−1154.633 661	T	cumulative
3.5	3	−1154.633 657	T	cumulative
3.5	CAS	−1154.633 655	T	cumulative
4.5	0	−1154.669 490	T	cumulative
4.5	1	−1154.669 653	T	cumulative
4.5	1	−1154.669 651	T	local
4.5	2	−1154.669 655	T	cumulative
4.5	CAS	−1154.669 656	T	cumulative
5.0	0	−1154.671 701	T	cumulative
5.0	1	−1154.671 740	T	cumulative
5.0	1	−1154.671 739	T	local
5.0	2	−1154.671 740	T	cumulative
5.0	CAS	−1154.671 740	T	cumulative
6.0	0	−1154.673 126	T	cumulative
6.0	1	−1154.673 125	T	cumulative
6.0	1	−1154.673 130	T	local
6.0	2	−1154.673 128	T	cumulative
6.0	CAS	−1154.673 128	T	cumulative
20.0	0	−1154.674 761	T	cumulative
20.0	CAS	−1154.674 795	T	cumulative
3.0	0	−1154.483 846	F	cumulative
3.0	1	−1154.499 365	F	cumulative
3.0	2	−1154.499 811	F	cumulative
3.0	3	−1154.499 810	F	cumulative
3.0	CAS	−1154.500 000	F	cumulative
3.5	0	−1154.624 159	F	cumulative
3.5	1	−1154.628 593	F	cumulative
3.5	2	−1154.628 614	F	cumulative
3.5	3	−1154.628 631	F	cumulative
3.5	CAS	−1154.628 730	F	cumulative
4.5	0	−1154.668 048	F	cumulative
4.5	1	−1154.668 367	F	cumulative
4.5	2	−1154.668 363	F	cumulative
4.5	CAS	−1154.668 432	F	cumulative
5.0	0	−1154.670 944	F	cumulative
5.0	1	−1154.671 024	F	cumulative
5.0	2	−1154.671 024	F	cumulative
5.0	CAS	−1154.671 076	F	cumulative
6.0	0	−1154.672 910	F	cumulative
6.0	1	−1154.672 918	F	cumulative
6.0	2	−1154.672 918	F	cumulative
6.0	CAS	−1154.672 968	F	cumulative
20.0	0	−1154.674 755	F	cumulative
20.0	CAS	−1154.674 793	F	cumulative

Table 2: Results for the N_4 tetrahedron.

	Defined				Empirical
Algorithm	S	S_z	n_{exc}	E/E_{h}	S
Davidson	0		CAS	−217.691 055	
Davidson	1		CAS	−217.690 423[a]	
Davidson	2		CAS	−217.689 159	
Davidson	3		CAS	−217.687 262	
Davidson	4		CAS	−217.684 734	
Davidson	5		CAS	−217.681 574	
Davidson	6		CAS	−217.677 788	
Davidson	0		0	−217.669 068	
Davidson	1		0	−217.669 443	
Davidson	2		0	−217.670 281	
Davidson	3		0	−217.671 504	
Davidson	4		0	−217.673 192	
Davidson	5		0	−217.675 255	
Davidson	6		0	−217.677 788	
Davidson	0		1	−217.690 918	
Davidson	1		1	−217.690 300	
Davidson	2		1	−217.689 063	
Davidson	3		1	−217.687 202	
Davidson	4		1	−217.684 709	
Davidson	5		1	−217.681 574	
Davidson	6		1	−217.677 788	
Davidson	0		2	−217.691 055	
Davidson	1		2	−217.689 963[b]	
Davidson	2		2	−217.689 159	
Davidson	3		2	−217.687 262	
Davidson	4		2	−217.684 734	
Davidson	5		2	−217.681 574	
Davidson	6		2	−217.677 788	
FCIQMC		0	CAS	−217.690 927	0.017 992
FCIQMC		1	CAS	−217.690 294	0.999 335
FCIQMC		2	CAS	−217.689 108	2.000 011
FCIQMC		3	CAS	−217.687 259	2.999 996
FCIQMC		4	CAS	−217.684 734	4.000 000
FCIQMC		5	CAS	−217.681 574	5.000 000
FCIQMC		6	CAS	−217.677 788	6.000 000
FCIQMC		0	1	−217.690 805	0.014 956
FCIQMC		1	1	−217.690 190	0.998 939
FCIQMC		2	1	−217.689 025	2.000 015
FCIQMC		3	1	−217.687 200	3.000 002
FCIQMC		4	1	−217.684 709	4.000 000
FCIQMC		5	1	−217.681 574	5.000 000
FCIQMC		6	1	−217.677 788	6.000 000
FCIQMC		0	2	−217.690 927	0.018 142
FCIQMC		1	2	−217.690 294	1.001 640
FCIQMC		2	2	−217.689 111	2.000 047
FCIQMC		3	2	−217.687 259	2.999 990
FCIQMC		4	2	−217.684 734	4.000 000
FCIQMC		5	2	−217.681 574	5.000 000
FCIQMC		6	2	−217.677 788	6.000 000

[a] Davidson CI optimization was in a local minimum and had to be restarted with the CI-vector of the Davidson GAS $n_{\text{exc}} = 1$ calculation.

[b] Davidson CI optimization was in a local minimum. For technical reasons it could not be restarted from converged CI-solutions.

Table 3: Results for the Fe_4S_4 cluster. The spin-pure GUGA results are taken from reference [12].

basis	S	n_{exc}	E/E_h	$\sigma(E)/\text{m}E_\text{h}$	$(E - E(\text{GUGA FCI}))/\text{m}E_\text{h}$
slater	0	1	-8432.54411810	0.00080	1.05553
slater	0	2	-8432.54459286	0.00071	0.58077
slater	0	CAS	-8432.54459396	0.00079	0.57967
slater	1	1	-8432.54311601	0.00755	1.51837
slater	1	2	-8432.54350770	0.00791	1.12668
slater	1	CAS	-8432.54351238	0.00577	1.12200
slater	2	1	-8432.54200918	0.08352	1.57917
slater	2	2	-8432.54224563	0.03242	1.34272
slater	2	CAS	-8432.54225342	0.07292	1.33493
slater	3	1	-8432.54095658	0.13840	1.11660
slater	3	2	-8432.54123450	0.15041	0.83868
slater	3	CAS	-8432.54134563	0.10314	0.72755
slater	4	1	-8432.53964877	0.07697	0.52708
slater	4	2	-8432.53982452	0.09787	0.35133
slater	4	CAS	-8432.53993122	0.04333	0.24463
slater	5	1	-8432.53765165	0.04632	0.30189
slater	5	2	-8432.53777222	0.02443	0.18132
slater	5	CAS	-8432.53779243	0.02172	0.16111
slater	6	1	-8432.53525882	0.01031	0.21119
slater	6	2	-8432.53551695	0.04561	-0.04694
slater	6	CAS	-8432.53550026	0.02918	-0.03025
slater	7	1	-8432.53272305	0.02581	0.06260
slater	7	2	-8432.53275652	0.04259	0.02913
slater	7	CAS	-8432.53278919	0.00508	-0.00354
slater	8	1	-8432.52997451	0.00143	0.02600
slater	8	2	-8432.52999146	0.00161	0.00905
slater	8	CAS	-8432.52996841	0.00248	0.03210
slater	9	1	-8432.52720956	0.00044	0.00510
slater	9	2	-8432.52721032	0.00032	0.00434
slater	9	CAS	-8432.52722031	0.00031	-0.00565
slater	10	1	-8432.52452663	0.00000	0.00025
slater	10	2	-8432.52452663	0.00000	0.00025
slater	10	CAS	-8432.52452663	0.00000	0.00025
GUGA	0	CAS	-8432.54517363		0
GUGA	1	CAS	-8432.54463438		0
GUGA	2	CAS	-8432.54358835		0
GUGA	3	CAS	-8432.54207318		0
GUGA	4	CAS	-8432.54017585		0
GUGA	5	CAS	-8432.53795354		0
GUGA	6	CAS	-8432.53547001		0
GUGA	7	CAS	-8432.53278565		0
GUGA	8	CAS	-8432.53000051		0
GUGA	9	CAS	-8432.52721466		0
GUGA	10	CAS	-8432.52452688		0

3 Evaluate $\hat{S}^2$ with 2-RDM

The aim is to bring the operator into the same format as the reduced density matrices. We start with

$$\hat{S}^2 = \hat{S}_+\hat{S}_- + \hat{S}_z\left(\hat{S}_z - 1\right) \tag{3.1}$$

and

$$\begin{aligned}
\hat{S}_+ &= \sum_p a^\dagger_{p\alpha} a_{p\beta} \\
\hat{S}_- &= \sum_p a^\dagger_{p\beta} a_{p\alpha} \\
\hat{S}_z &= \frac{1}{2}\sum_p \left(a^\dagger_{p\alpha} a_{p\alpha} - a^\dagger_{p\beta} a_{p\beta}\right)
\end{aligned} \tag{3.2}$$

We evaluate:

$$\hat{S}_+\hat{S}_- = \sum_p a^\dagger_{p\alpha} a_{p\alpha} - \sum_{pq} a^\dagger_{p\alpha} a^\dagger_{q\beta} a_{p\beta} a_{q\alpha} \tag{3.3}$$

and

$$\begin{aligned}
4\hat{S}_z\left(\hat{S}_z - 2\right) = &\sum_{pq}\left(a^\dagger_{p\alpha} a_{p\alpha} a^\dagger_{q\alpha} a_{q\alpha} - a^\dagger_{p\alpha} a_{p\alpha} a^\dagger_{q\beta} a_{q\beta} - a^\dagger_{p\beta} a_{p\beta} a^\dagger_{q\alpha} a_{q\alpha} + a^\dagger_{p\beta} a_{p\beta} a^\dagger_{q\beta} a_{q\beta}\right) \\
&- 2\sum_p \left(a^\dagger_{p\alpha} a_{p\alpha} - a^\dagger_{p\beta} a_{p\beta}\right) \quad .
\end{aligned} \tag{3.4}$$

We note the following identities:

$$\begin{aligned}
a^\dagger_{p\alpha} a_{p\alpha} a^\dagger_{q\alpha} a_{q\alpha} &= \delta^{pq} a^\dagger_{p\alpha} a_{q\alpha} - a^\dagger_{p\alpha} a^\dagger_{q\alpha} a_{p\alpha} a_{q\alpha} \\
-a^\dagger_{p\alpha} a_{p\alpha} a^\dagger_{q\beta} a_{q\beta} &= -\underbrace{\delta^{p\alpha q\beta} a^\dagger_{p\alpha} a_{q\beta}}_{0} + a^\dagger_{p\alpha} a^\dagger_{q\beta} a_{p\alpha} a_{q\beta} = a^\dagger_{p\alpha} a^\dagger_{q\beta} a_{p\alpha} a_{q\beta} \\
-a^\dagger_{p\beta} a_{p\beta} a^\dagger_{q\alpha} a_{q\alpha} &= -\underbrace{\delta^{p\beta q\alpha} a^\dagger_{p\beta} a_{q\alpha}}_{0} + a^\dagger_{p\beta} a^\dagger_{q\alpha} a_{p\beta} a_{q\alpha} = a^\dagger_{p\beta} a^\dagger_{q\alpha} a_{p\beta} a_{q\alpha} \\
a^\dagger_{p\beta} a_{p\beta} a^\dagger_{q\beta} a_{q\beta} &= \delta^{pq} a^\dagger_{p\beta} a_{q\beta} - a^\dagger_{p\beta} a^\dagger_{q\beta} a_{p\beta} a_{q\beta}
\end{aligned} \tag{3.5}$$

Now we can write:

$$\begin{aligned}
4\hat{S}_z\left(\hat{S}_z - 2\right) = &-\sum_p a^\dagger_{p\alpha} a_{p\alpha} + 3\sum_p a^\dagger_{p\beta} a_{p\beta} \\
&- \sum_{p\neq q}\left(a^\dagger_{p\alpha} a^\dagger_{q\alpha} a_{p\alpha} a_{q\alpha} + a^\dagger_{p\beta} a^\dagger_{q\beta} a_{p\beta} a_{q\beta}\right) \\
&+ \sum_{pq}\left(a^\dagger_{p\alpha} a^\dagger_{q\beta} a_{p\alpha} a_{q\beta} + a^\dagger_{p\beta} a^\dagger_{q\alpha} a_{p\beta} a_{q\alpha}\right) \quad .
\end{aligned} \tag{3.6}$$

We conclude:

$$\begin{aligned}\hat{S}^2 &= \hat{S}_+\hat{S}_- + \hat{S}_z\Big(\hat{S}_z - 1\Big) \\ &= \frac{3}{4}\sum_p \Big(a^\dagger_{p\alpha}a_{p\alpha} + a^\dagger_{p\beta}a_{p\beta}\Big) \\ &\quad - \frac{1}{4}\sum_{p\neq q}\Big(a^\dagger_{p\alpha}a^\dagger_{q\alpha}a_{p\alpha}a_{q\alpha} + a^\dagger_{p\beta}a^\dagger_{q\beta}a_{p\beta}a_{q\beta}\Big) \\ &\quad + \frac{1}{4}\sum_{pq}\Big(2a^\dagger_{p\alpha}a^\dagger_{q\beta}a_{p\alpha}a_{q\beta} - 4a^\dagger_{p\alpha}a^\dagger_{q\beta}a_{p\beta}a_{q\alpha}\Big)\end{aligned} \tag{3.7}$$

References

(1) Widmark, P.-O.; Malmqvist, P.-Å.; Roos, B. O. *Theoret. Chim. Acta* **1990**, *77*, 291–306.

(2) Roos, B. O.; Lindh, R.; Malmqvist, P.-Å.; Veryazov, V.; Widmark, P.-O. *J. Phys. Chem. A* **2004**, *108*, 2851–2858.

(3) Aquilante, F.; Pedersen, T. B.; Lindh, R. *J. Chem. Phys.* **2007**, *126*, 194106.

(4) Aquilante, F.; Lindh, R.; Bondo Pedersen, T. *J. Chem. Phys.* **2007**, *127*, 114107.

(5) Aquilante, F.; Pedersen, T. B.; Lindh, R.; Roos, B. O.; Sánchez de Merás, A.; Koch, H. *J. Chem. Phys.* **2008**, *129*, 024113.

(6) Aquilante, F.; Gagliardi, L.; Pedersen, T. B.; Lindh, R. *J. Chem. Phys.* **2009**, *130*, 154107.

(7) Pedersen, T. B.; Aquilante, F.; Lindh, R. *Theor Chem Acc* **2009**, *124*, 1–10.

(8) Weser, O.; Freitag, L.; Guther, K.; Alavi, A.; Li Manni, G. *Int. J. Quantum Chem.* **2021**, *121*, 26454–26467.

(9) Li Manni, G.; Alavi, A. *J. Phys. Chem. A* **2018**, *122*, 4935–4947.

(10) Guther, K. et al. *J. Chem. Phys.* **2020**, *153*, 34107–34131.

(11) Fdez. Galván, I. et al. *J. Chem. Theory Comput.* **2019**, *15*, 5925–5964.

(12) Dobrautz, W.; Weser, O.; Bogdanov, N.; Alavi, A.; Manni, G. L. *arXiv* **2021**, *2106.07775.*
